# Strong Metal‐Support Interactions on TiO_2_‐Supported Metal Catalysts for Fine‐Tuning Catalysis

**DOI:** 10.1002/anie.202502611

**Published:** 2025-11-03

**Authors:** Joby Sebastian, Chalachew Mebrahtu, Feng Zeng, Regina Palkovits

**Affiliations:** ^1^ Chair of Heterogeneous Catalysis and Technical Chemistry Institute for Technical and Macromolecular Chemistry RWTH Aachen University Worringerweg 2 52074 Aachen Germany; ^2^ Institute for a Sustainable Hydrogen Economy Forschungszentrum Jülich GmbH Marie‐Curie‐Str. 5 52428 Jülich Germany; ^3^ State Key Laboratory of Materials‐Oriented Chemical Engineering College of Chemical Engineering Nanjing Tech University Nanjing Jiangsu 211816 China; ^4^ Max‐Planck‐Institut for Chemical Energy Conversion Stiftstr. 34–36 45470 Mülheim an der Ruhr Germany

**Keywords:** Encapsulation, Heterogeneous catalysis, Metal‐support interactions, Titania

## Abstract

The concept of strong metal‐support interaction (SMSI), a topic of investigation for over four decades, remains an important area of research in heterogeneous catalysis. While substantial progress has been made in understanding SMSI across various metal/support systems, its complexity continues to evolve. TiO_2_, recognized as the first support material to exhibit SMSI, has been instrumental in advancing this understanding. However, the lack of a comprehensive guide on how various factors influence SMSI on TiO_2_ has limited the ability to systematically control these interactions. This review seeks to provide a consolidated view of the current understanding of SMSI in TiO_2_‐supported metal catalysts. It highlights the sensitivity of SMSI on TiO_2_ to a range of parameters, including pretreatment conditions and reaction environments. The dynamic nature of SMSI under reaction conditions is also explored, underscoring its complexity and potential for fine‐tuning catalyst performance. Additionally, practical insights into innovative chemical methods for controlling SMSI on TiO_2_ are discussed, offering strategies for modulating SMSI to meet specific catalytic requirements. By addressing these aspects, this review aims to offer a valuable guide for the rational design of metal/TiO_2_ catalysts, ultimately contributing to a more refined and controllable approach to SMSI on TiO_2_.

## Graphical abstract

This review offers an in‐depth overview and guide to the origin, modulation, and dynamics of strong metal‐support interaction (SMSI) in TiO_2_‐based catalysts for optimizing catalysis. It explores the functions of oxygen vacancies, surface/alloy formation energies, and TiO_2_ structural/thermal aspects. The benefits of pre‐SMSI conditions, the changing dynamics of SMSI in catalysis, challenges, and future directions are highlighted.

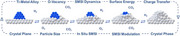

## 1 Introduction

Metal‐support interactions (MSI) encompass various phenomena including charge transfer, interfacial effects, nanoparticle crystal structure and morphology, chemical composition, and SMSI.^[^ ^1^, ^2^, ^3^, ^4^, ^5^ ^]^ Among these, SMSI has emerged as a critical catalyst design parameter in heterogeneous catalysis. Classical SMSI (C‐SMSI) is characterized by the encapsulation of metal nanoparticles, such as those from the platinum group (Ru, Rh, Pd, Ir, and Pt)‐by their partially reduced metal oxide supports, including TiO_2_,^[^ ^6^, ^7^ ^]^ CeO_2_,^[^ ^8^, ^9^, ^10^ ^]^ Nb_2_O_5_,^[^ ^11^, ^12^ ^]^ Fe_2_O_3_,^[^ ^13^, ^14^ ^]^ V_2_O_3_,^[^ ^15^, ^16^ ^]^ ZnO,^[^ ^17^, ^18^ ^]^ and Ta_2_O_5_.^[^ ^19^ ^]^ This encapsulation typically occurs after high‐temperature treatment (>400 °C) in a reducing atmosphere such as H_2_. It is reversible at lower temperatures (<400 °C) under oxidizing conditions such as O_2_, where the encapsulated metal oxide reoxidizes and reintegrates into the bulk oxide support.^[^ ^1^, ^4^, ^5^ ^]^ The advantages of C‐SMSI include the simplicity of needing only H_2_ as a reagent and the control afforded by a single‐step process. Moreover, the byproduct of C‐SMSI is water making it a green route for SMSI generation.

Recent advancements have expanded the understanding of SMSI to include nonclassical manifestations. Generally, any approach that generates materials with physico–chemical properties analogous to C‐SMSI, without relying on high‐temperature reduction under H_2_, is classified as nonclassical SMSI.^[^ ^20^ ^]^ These approaches include wet chemical methods that rely on various reagents,^[^ ^21^ ^]^ reactant or adsorbate‐induced,^[^ ^10^, ^22^, ^23^ ^]^ laser‐induced,^[^ ^24^ ^]^ and atomic layer deposition techniques.^[^ ^25^, ^26^ ^]^ Nonclassical SMSI aims to preserve metal nanoparticle size and maximize its surface exposure. Moreover, the concept of SMSI now extends beyond traditional reducible oxides to include hydroxyapatite,^[^ ^27^ ^]^ and Group 1B metals such as Cu,^[^ ^28^, ^29^ ^]^ Ag,^[^ ^30^, ^31^ ^]^ and Au.^[^ ^32^, ^33^ ^]^ More specifically, nonclassical SMSI have been classified as oxidative SMSI (O‐SMSI), adsorbate‐mediated SMSI (A‐SMSI), reactive metal‐support interaction (RMSI), wet‐chemistry SMSI (WC‐SMSI), electronic oxide‐metal strong interaction (EOMSI), metal carbide‐induced SMSI (MC‐SMSI), and SMSI between single atoms and supports.^[^ ^5^ ^]^ Whether it is classical or nonclassical SMSI, the underlying thermodynamics remain consistent except the difference of the method used for the initial reduction of the support material.

Thermodynamically, the driving force for C‐SMSI during high‐temperature treatment is the minimization of the system's overall free energy, which includes surface, interface, and bulk energies.^[^ ^34^ ^]^ For a bare support metal oxide, energy minimization is limited to surface and bulk contributions. In metal oxide‐supported nanoparticles, free energy minimization can occur through mechanisms such as nanoparticle growth, support crystallization, support phase transformation, and alloy/intermetallic formation between the nanoparticles and the metal oxide support. A carefully controlled high‐temperature treatment, where crystallization and nanoparticle growth (because of their Tammann temperatures) are minimized, leads primarily to the minimization of metal surface and interface energies. This optimal condition facilitates the gradual decoration of metal nanoparticles with partially reduced support metal oxide atoms, eventually leading to complete encapsulation. Specifically, the initial step involves the metal‐assisted reduction of the support material via a hydrogen spillover mechanism. Given that most support metal oxides reduce relatively at temperatures above 400 °C, this temperature condition becomes essential for initiating C‐SMSI.

While SMSI, whether classical or nonclassical, leads to a reduction in the number of exposed surface atoms on metal nanoparticles, this phenomenon confers significant advantages to the catalytic properties of the material as a whole. These include: 1) an increase in the intrinsic activity of the exposed metal atoms, driven by geometric effects (surface decoration resulting in ligand effects).^[^ ^1^, ^5^ ^]^ 2) a pronounced or complete shift in selectivity, resulting from the dynamic nature of SMSI generating new interfaces and blocking specific active sites,^[^ ^1^, ^5^ ^]^ and 3) a significant enhancement in stability by preventing nanoparticle sintering (low Tammann temperatures),^[^ ^5^, ^35^, ^36^ ^]^ particularly in reactions where catalytic performance is driven more by activity than selectivity.

Many recent reviews have been published, exploring various aspects of SMSI,^[^ ^5^, ^37^, ^38^, ^39^, ^40^, ^41^, ^42^, ^43^, ^44^, ^45^, ^46^, ^47^, ^48^, ^49^, ^50^ ^]^ including its influence on catalysis (activity, selectivity, and stability),^[^ ^5^, ^40^, ^44^, ^47^ ^]^ insights gained through in situ TEM studies,^[^ ^37^, ^49^ ^]^ SMSI on atomic, cluster and nanoparticle catalysis,^[^ ^50^ ^]^ comprehensive summaries of different SMSI types, and its role in environmental catalysis.^[^ ^38^ ^]^ Other key topics discussed include the impact of gas environments on SMSI,^[^ ^41^ ^]^ its role in Fischer–Tropsch synthesis,^[^ ^42^ ^]^ and adsorbate‐induced SMSI.^[^ ^43^ ^]^ Additionally, O‐SMSI,^[^ ^45^ ^]^ nonclassical SMSI,^[^ ^20^ ^]^ and its applications in photocatalysis^[^ ^46^ ^]^ and electrocatalysis^[^ ^39^, ^48^ ^]^ have been extensively explored. Each of these reviews covers a diverse range of support materials, such as CeO_2_, TiO_2_, ZnO, Fe_2_O_3_, MoO_3_, Ga_2_O_3_, In_2_O_3_, hydroxyapatite, and others. Although these reviews emphasize the significance, advancements, and benefits of SMSI during catalysis, there is a notable gap in the literature dedicated to detailed discussion exclusively on specific support materials. Targeted investigations of existing studies on individual supports are essential to identify the key factors that govern SMSI induction, strength, and dynamics under varying reaction conditions specific to that support. By narrowing the scope to a single support, researchers can better predict SMSI behavior and select optimal parameters for desired outcomes. Since SMSI varies across different supports, this targeted approach enhances control over catalytic performance and enables a more rational catalyst design.

Given the extensive use of TiO_2_ in various catalytic reactions, its distinction as the first support to exhibit SMSI,^[^ ^6^, ^7^ ^]^ and the critical role SMSI plays in shaping its catalytic behavior, a comprehensive guide of SMSI behavior and the factors influencing TiO_2_‐based systems is of significant importance, yet remains lacking. Such a guide would be invaluable in designing targeted metal/TiO_2_ catalysts for various chemical transformations. The unique properties of TiO_2_,^[^ ^51^, ^52^, ^53^, ^54^, ^55^, ^56^, ^57^, ^58^, ^59^, ^60^, ^61^ ^]^ such as its rich surface chemistry with a high density of oxygen vacancies and its redox properties, significantly affect SMSI. These characteristics enable dynamic interactions with metal particles, including reversible nanoparticle encapsulation. Additionally, the polymorphic forms of TiO_2_, like anatase and rutile, further influence SMSI behavior. By investigating these kinds of interactions, a deeper understanding of how SMSI evolves on metal/TiO_2_ and how it can be tuned for enhanced catalytic performance (activity, selectivity, and stability) can be achieved. This review would provide a predictive framework for optimizing SMSI on TiO_2_ through the modulation of crystalline phases, morphologies, thermal treatments, and reaction conditions, both individually and simultaneously, under oxidizing and reducing environments.

This predictive framework is built upon a thorough examination of the complex and multifaceted nature of SMSI on TiO_2_. This review begins by illustrating the generation and reversibility of TiO_2_‐based SMSI, as revealed by in situ studies. Critical parameters influencing SMSI, such as oxygen vacancies, metal surface energy, alloy formation, Ti affinity of metals, crystalline phase, facet dependency, reduction and calcination temperatures, and particle size are analyzed. Additionally, several innovative chemical methods for regulating SMSI on TiO_2_‐based systems are briefly outlined. The dynamic behavior of SMSI is also highlighted, emphasizing that it is not merely an ex situ factor for performance enhancement but a highly dynamic phenomenon influenced by reaction conditions. Finally, we provide a perspective that encompasses the advantages of understanding the complexity and variability of SMSI on TiO_2_ and its potential to develop more efficient and adaptable catalytic systems.

## 2 Observing C‐SMSI: Origins, Evolution, Reversibility, and Structural Transformations

**Figure 1:**
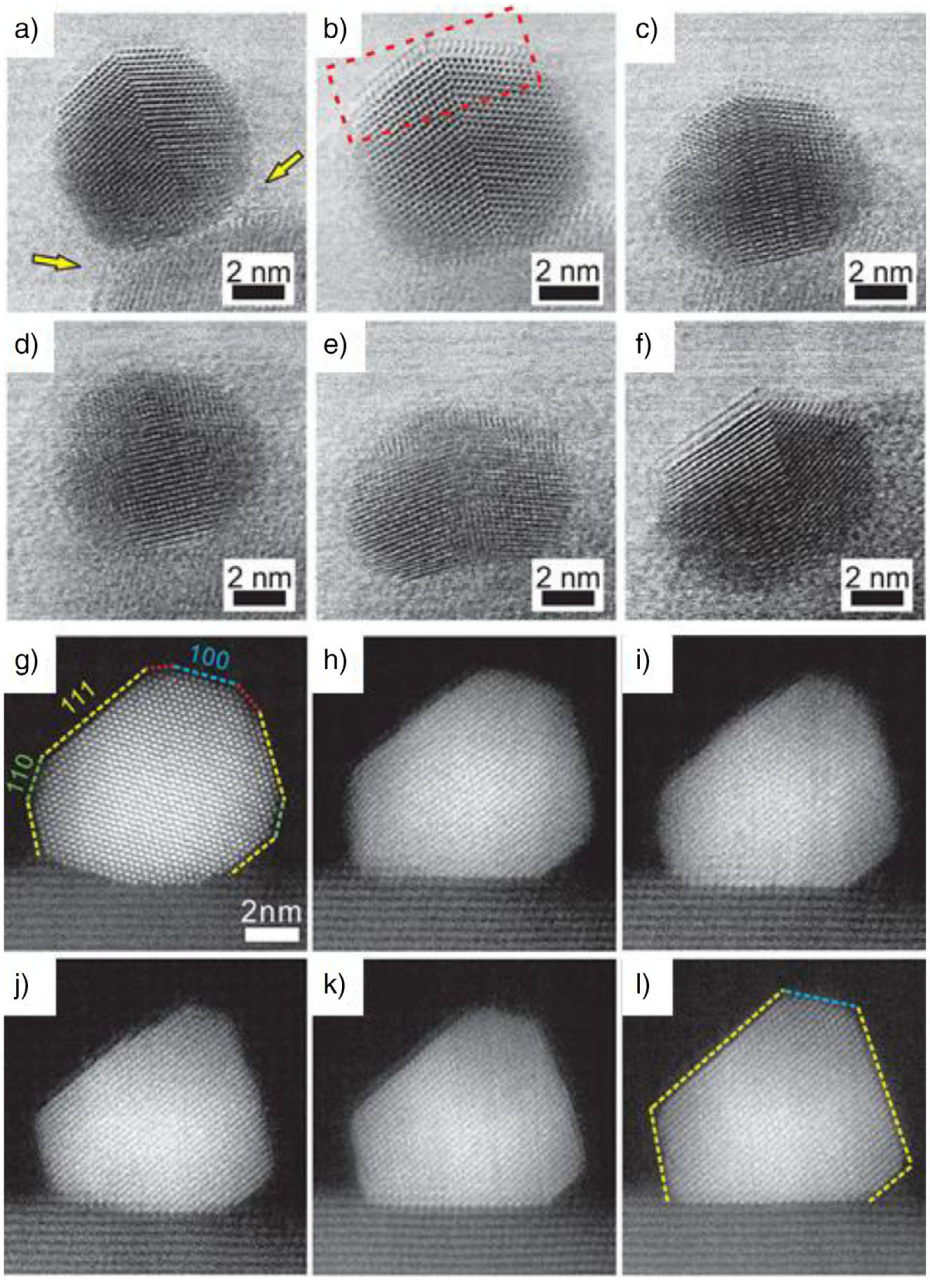
Sequential in situ observations of reversible SMSI layer formation on Pd nanocrystal in Pd/TiO_2_‐P25: a) under H_2_/Ar at 1 atm at 250 °C; b). then 500 °C for 10 min; c) under O_2_ (150 Torr) at 250 °C for 8 min; d) then 15 min; e) stable state at 500 °C; f) finally, under H_2_/Ar at 1 atm at 500 °C for 5 min. g–l) Dynamic shape change of Pd nanocrystal during SMSI: g) under H_2_ (4.9 vol%)/O_2_ (2 vol%)/Ar 1 atm at 400 °C; h) 400 °C for 25 min; i) 400 °C for 30 min; j) 500 °C for 3 min; k) 500 °C for 22 min. l) Final stable state at 500 °C. Reprinted with permission from Ref. [63]. Copyright 2016, American Chemical Society.

The static nature of SMSI can be effectively analyzed using ex situ transmission electron microscopy (TEM). However, this approach only captures a snapshot of SMSI without revealing its time‐dependent evolution or progression. Recent advancements in situ TEM techniques have significantly enhanced the understanding of supported metal catalysts by providing insights into their structural evolution and behavior under varying temperatures and reaction conditions.^[^ ^62^ ^]^ An in situ TEM study on Pd/TiO_2_‐P25 (P25‐contains both anatase and rutile phases) has traced the reversible nature of SMSI and the structural changes it induces during this process.^[^ ^63^ ^]^ It was observed that even at a relatively low temperature of 250 °C under H_2_ environment, an amorphous TiO_x_ layer was detected to form on Pd, originating from the interface between Pd and TiO_2_‐P25 (Figure 1a). As the temperature increased to 500 °C, the amorphous layer began to crystallize, eventually forming a bilayer that appeared epitaxial with the Pd(111) plane (Figure 1b) and covered nearly 90% of the Pd nanoparticles on TiO_2_‐P25. According to the density functional theory (DFT) calculations, this bilayer consists of a Ti_2_O_3_ phase containing titanium in the + 3 oxidation state. The calculations also show that when Pt is used instead of Pd, the overlayer remains stable. However, with Au, the overlayer becomes unstable. The SMSI is governed by the nature of the metal on TiO_2_. When the gas environment shifted from H_2_ to O_2_, the crystalline overlayer became amorphous and eventually dissipated, exposing the Pd nanoparticles (Figure 1c–e). However, this overlayer reformed when the H_2_ atmosphere was restored (Figure 1f). These observations highlight the origin, evolution, and reversibility of SMSI on TiO_2_.

Furthermore, the shape of the Pd nanoparticles was found to be influenced by the formation of SMSI.^[^ ^63^ ^]^ In the absence of an overlayer, the nanoparticles exhibited a truncated octahedron shape with rounded corners (Figure 1g). With a single TiO_x_ overlayer, the (111) facets of Pd expanded while the (100) facets contracted, and the (110) and other high‐index facets were lost (Figure 1h–l). When a double layer formed, the ratio of (111) to (100) planes of Pd increased from 1.08 for the monolayer to 1.27 for the double layer. The shape change, driven by the minimization of surface energy due to the (111) plane having the lowest surface energy, occurred through the migration of Pd atoms between the (111) and (100) facets. Overall, SMSI on Pd(Pt)/TiO_2_‐P25 not only results in encapsulation but also induces significant alterations in the shape of the nanoparticles. These findings provide valuable insights into the origin, evolution, reversibility, and structural changes of C‐SMSI on TiO_2_.

## 3 Tracing the Roots: Decoding the Critical Roles of Oxygen Vacancies, Surface Energy, and Alloy Formation

Identifying the source factors that induce SMSI is crucial for successfully controlling it. Early studies with Pt/TiO_2_(100)‐R (R designates rutile) and Nb‐doped Pt/Nb_x_Ti_1‐x_O_2_(100)‐R identified two key factors responsible for the encapsulation: 1) bulk oxygen vacancies, which act as a source, and 2) the surface free energy of the metal nanoparticles, which serves as the driving force.^[^ ^64^ ^]^ It was noticed that the encapsulation occurs more quickly for Pt on nonstoichiometric TiO_2_ (contains oxygen vacancies), requiring only 3 min of annealing at 500 °C, whereas the same process takes 15 min on stoichiometric TiO_2_ due to less amount of oxygen vacancies. Nb substitution for Ti in the rutile lattice of TiO_2_ significantly stabilizes the (100) surface. Since oxygen vacancies are less able to diffuse to a stable surface, the interface becomes more inert to TiO_x_ suboxide formation, and thus, SMSI is less likely to occur. These findings emphasize the crucial role of oxygen vacancies and their diffusion from the bulk to the surface during the encapsulation process.

The driving force for SMSI comes from the surface energy of the metal nanoparticles. To minimize surface and interface energy, encapsulation through SMSI becomes the thermodynamically most probable outcome. The surface free energy of common metals based on their enthalpies of atomization follows the specific order: Os (3.9 J m^−^^2^) > Ru (3.4 J m^−^^2^) > Rh (2.8 J m^−^^2^) > Pt (2.7 J m^−^^2^) > Co (2.7 J m^−^^2^) > Ni (2.4 J m^−^^2^) > Pd (2.0 J m^−^^2^) > Cu (1.9 J m^−^^2^) > Au (1.6 J m^−^^2^) > Ag (1.3 J m^−^^2^).^[^ ^64^ ^]^ The higher the surface energy, the greater the tendency for encapsulation. This could be the reason why, in the above study,^[^ ^63^ ^]^ the overlayer became unstable when Pd was replaced by Au. Consequently, surface‐free energy can offer a useful approximation of the extent of encapsulation. In summary, both bulk oxygen vacancies (the source) and high metal surface energy (the driving force) are crucial for SMSI to take place. The interplay between bulk oxygen vacancies and surface free energy governs the extent of SMSI on a given metal and TiO_2_ and can be considered sensible descriptors for SMSI formation.

Building on this development, later studies further refined the understanding of SMSI by elaborating the above two key factors: 1) the electronic factor (oxygen vacancies) accompanied by Ti^n+^ diffusion and 2) the geometric factor (metal surface energy).^[^ ^65^ ^]^

### 3.1 The Electronic Factor

It involves charge transfer between the metal and the support. This is especially evident in TiO_2_, where high‐temperature reduction in H_2_ creates oxygen vacancies (the source) containing trapped electrons and interstitial Ti ions, making the TiO_2_ essentially electron‐rich. For instance, rutile TiO_2_, which normally has a 3.05 eV band gap, experiences a decrease to about 2.3 eV upon reduction.^[^ ^65^ ^]^ This reduction can create a shallow conduction band and move the Fermi level (E_F_) closer to the conduction band. When a metal with a higher work function (ϕ_metal_) than TiO_2_ (ϕ_TiO2_) is brought into contact with TiO_2_ (E_FTiO2_ > E_Fmetal_) the metal's Fermi level falls below the donor states of TiO_2_, causing charge transfer from TiO_2_ to the metal and resulting in a negative charge on the metal. For instance, in Pd/TiO_2_ systems, Pd becomes negatively charged because the work function of Pd (ϕ_Pd_ ≈ 5.6 eV) is higher than that of TiO_2_ (ϕ_TiO2_ ≈ 5.2 eV). This electron donation has been widely observed in X‐ray photoelectron spectroscopy (XPS) analyses of SMSI systems, where metals often gain a partial negative charge.

### 3.2 Diffusion of Ti^n+^ from Bulk to the Surface

**Figure 2:**
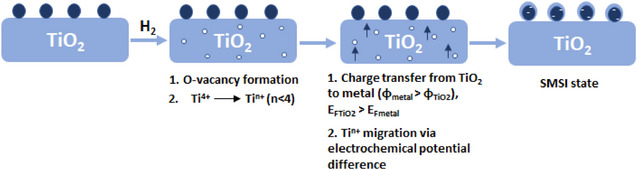
Illustration of key steps leading to the formation of the SMSI state: oxygen vacancy formation; titanium reduction; charge transfer and migration of Ti^n+^.

The diffusion of interstitial Ti^n+^ (n < 4), which was produced during the reduction, from the bulk of TiO_2_ to its surface occurs because of the difference in the electrochemical potential of Ti^n+^ across the phase boundary (Figure 2).^[^ ^65^ ^]^ The Ti^n+^ has high diffusivity at elevated temperatures (> 220 °C). On the other hand, the outward motion of O^2−^ anions is slow at these lower temperatures. Over time, this imbalance leads to the enrichment of Ti^n+^ on the surface, making the extent of TiO_2_ reduction crucial to this process.

### 3.3 The Geometric Factor

**Figure 3:**
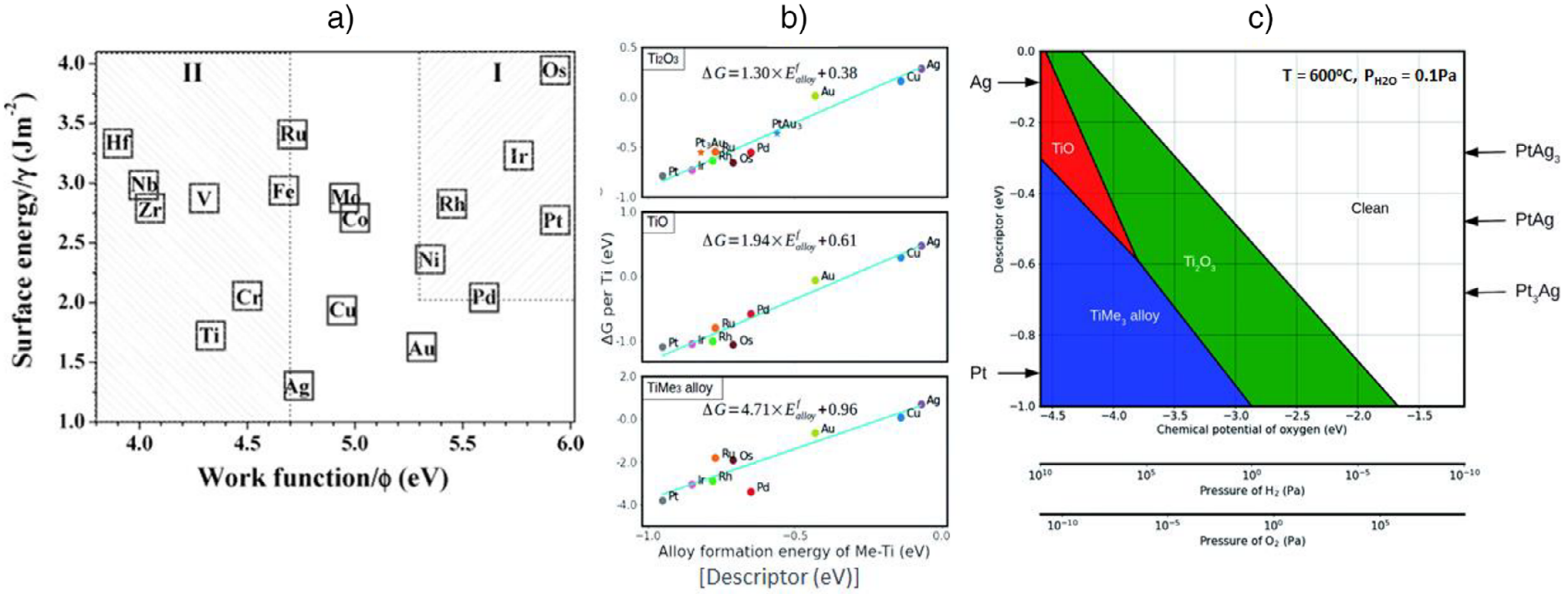
a) Correlation between the work function and surface energy of metals, indicating that higher values of both properties promote SMSI. Reprinted with permission from Ref. [65]. Copyright 2005, American Chemical Society. b) Relative stability of reduced TiO_x_ monolayers (Ti_2_O_3_, TiO, and TiMe_3_ alloy) on various metal (111) surfaces, shown as a function of Ti–Me alloy formation energy. Reprinted with permission from Ref. [34]. Copyright 2020, Royal Society of Chemistry. c) Phase diagram depicting the preferred monolayers on different metal (111) surfaces, depending on external environmental conditions (H_2_ and O_2_) and the descriptor at 600 °C. Reprinted with permission from Ref. [34]. Copyright 2020, Royal Society of Chemistry.

This involves the mass transport of Ti^n+^ from the TiO_2_ surface to the metal particles, leading to the encapsulation of the metal by a thin TiO_x_ layer. Several factors influence this encapsulation:^[^ ^65^ ^]^ a) support surface energy; supports with low surface energy (e.g., TiO_2_) are more prone to SMSI compared to those with higher surface energy (e.g., Al_2_O_3_). b) Metal surface energy; metals with high surface energy, such as Rh and Pt, favor encapsulation, as minimizing surface energy is a major driving force. c). Subsurface nonstoichiometry of TiO_2_; oxygen vacancies created by high‐temperature reduction enhance the likelihood of SMSI. d) Metal–metal versus metal–oxide interactions:^[^ ^66^ ^]^ If the metal–metal interaction (e.g., Pt–Pt) is stronger than the metal–oxide interaction (e.g., Pt–TiO_2_), the wetting ability of the metal is diminished, causing it to remain unspread on TiO_2_. This creates favorable conditions for the encapsulation of the metal by TiO_2_. Conversely, if the metal–oxide interaction is stronger, it can result in the formation of a metal–oxide species (e.g., PtO_x_) at the interface. This formation may hinder encapsulation by promoting the migration of oxygen over titanium, which can stabilize the metal–oxide bond and prevent metal atoms from being encapsulated within the oxide. The balance between these interactions affects encapsulation tendencies. For TiO_2_ support, under a specific set of treatment conditions, the sole parameters governing encapsulation are the surface free energy of the metals and the metal–metal versus metal–oxide interactions. In summary, the nonstoichiometric structure of the support, the metal's work function, metal's surface energy, and metal–metal bonding are all critical factors in determining SMSI behavior. A strong correlation exists between a metal's surface energy and work function (Figure 3a), with metals that have higher values of both exhibiting a stronger tendency toward SMSI.

Subsequent studies aimed to identify a more dependable and direct descriptor for the strength/stability of TiO_x_ monolayers across various metals.^[^ ^34^ ^]^ To achieve this, partially reduced Ti_2_O_3_ and TiO monolayers (derived from the most stable facets of anatase (101) and rutile (110)) were placed on the (111) surfaces of metals such as Pt, Ir, Rh, Ru, Os, Pd, Au, Cu, and Ag. For comparison, a Ti‐Me_3_ (Me designates metal) alloy monolayer as the topmost layer of the metal surface was also considered. The oxidation states of Ti in Ti_2_O_3_, TiO, and Ti‐Me_3_ are + 3, +2, and 0, respectively. In the case of Ti_2_O_3_ and TiO layers on metal (111) surfaces, electron transfer occurs from the Ti cations to the metals, reflecting the electronic factor discussed earlier. However, when the stability of the Ti_2_O_3_ and TiO monolayers on metal (111) surfaces was compared to the extent of charge transfer to the different metals, no clear linear correlation was obtained. The same held for the Ti‐Me_3_ alloy layer, indicating that charge donation (work function) is not a direct descriptor for the strength/stability of SMSI across different metals, though it remains a useful approximation for understanding its origin. Likewise, no clear linear correlation was found between monolayer (Ti_2_O_3_, TiO, Ti‐Me_3_) stability and the surface energy of the metal (111) surfaces. This further implies that while surface energy can be a helpful approximation for predicting the driving force, it cannot be used to predict the strength or stability of SMSI.

Next, the formation energy of Ti–metal alloys was tested as a possible descriptor, and a linear correlation emerged between monolayer stability and the most stable alloy formation energy (Figure 3b).^[^ ^34^ ^]^ This finding suggests that the alloy formation energy between Ti and metals could serve as a reliable predictor for SMSI strength between Ti_2_O_3_, TiO, Ti‐Me_3_ and various metals. The slopes of the linear fits (Figure 3b) indicated that the more reduced the monolayer, the stronger the metal–titanium bond, with strength following the trend Ti‐Me_3_ > TiO > Ti_2_O_3_. At elevated reduction temperatures, where the complete reduction of Ti atoms is highly favorable, SMSI becomes more stable as it leads to alloy formation. Since alloy formation energies of Ti with different metals are readily available from databases, this descriptor allows for rapid prediction of SMSI strength. Additionally, the descriptor has been successfully applied to alloys such as Pt‐Au, Pt‐Ag, Cu‐Ag, and Pt‐Ir. However, it is less reliable for metals with significantly different atomic radii (e.g., Pd).

The stability of these monolayers (Ti_2_O_3_, TiO, Ti‐Me_3_) was further examined in different gas environments.^[^ ^34^ ^]^ On a Pt(111) surface, at 0 °C and when the partial pressure of H_2_ (P_H2_) exceeds 10^−2^ Pa, the Ti_2_O_3_ monolayer was identified as the first stable phase. For the Au(111) surface, however, the Ti_2_O_3_ layer becomes stable only at much higher temperatures, around 500 °C onward under 1 bar of H_2_. This observation aligns with an experimental study on Au/TiO_2_ using high‐resolution transmission electron microscopy (HRTEM), which demonstrated that at 500 °C under flowing 10% H_2_, Au becomes encapsulated by a TiO_x_ layer primarily composed of Ti^3+^.^[^ ^32^ ^]^ Overall, in addition to temperature, the stability of the monolayer is also strongly influenced by the P_H2_. As temperature and pressure increase, the monolayer stability progresses through a sequence: from a clean metal surface to Ti_2_O_3_, followed by TiO, and ultimately transitioning to a Ti‐Me_3_ alloy phase. A phase diagram (Figure 3c) depicting the behavior of these phases as a function of gas atmospheres (H_2_ and O_2_) at 600 °C offers a useful tool for selecting appropriate metals and environmental conditions to control SMSI on TiO_2_ for catalytic applications.

Molecular dynamics simulations have also been used to study SMSI and to identify a suitable descriptor for the probability and kinetics of SMSI‐induced encapsulation.^[^ ^67^ ^]^ For this, rutile TiO_2_(011)‐supported 305‐atom Pt, Pd, Rh, Ru, Cu, and Ag clusters were examined under elevated temperatures. Reduction conditions without H_2_ were modeled by introducing 12.5% oxygen vacancies. This led to the migration of TiO_2‐x_ suboxide onto Pt, Pd, Rh, and Ru but not onto Cu and Ag. This behavior was correlated with the metal–metal in oxide affinity term, denoted as Q(MM′), where M represents the metal (Pt, Pd, etc.) and M′ is Ti. Q(MM′) was calculated based on the enthalpy of alloy formation per mole of metal and Ti from gaseous metal atoms, making it a quantifiable parameter. The Q(MM′) value for Pt, Pd, Rh, and Ru was greater than 10.8 eV, whereas for Cu and Ag, it was less than 8.8 eV. A higher Q(MM′) value corresponded to a greater tendency for encapsulation. The simulations also showed that for Pt/TiO_2_, TiO_2‐x_ suboxide migration was accompanied by Pt‐Ti alloy formation, as some Ti atoms permeated into the Pt particles. However, for Rh/TiO_2_, no Ti permeation into Rh particles was observed, preventing alloy formation. The migration of TiO_2‐x_ overlayers onto metal particles depended on the diffusion coefficient (D_e_) of Ti, a quantifiable term. The advantage of D_e_ is that it can be used to quantify encapsulation kinetics. D_e_ was found to be linearly dependent on the Ti affinity (Q(MM′)) of the metals, with stronger Ti affinity leading to faster encapsulation kinetics. For example, Pt, with a Ti affinity of 13.0 eV, exhibited a D_e_ value approximately 20% higher than that of Pd, which had a Ti affinity of 10.8 eV. As a result, Pt demonstrated faster encapsulation kinetics than Pd. Although a high Ti affinity promotes TiO_2‐x_ migration onto metal particles, the diffusion of the formed TiO_2‐x_ layer on the metal surface is energetically unfavorable. Therefore, the rate‐determining step of the encapsulation process is the migration of TiO_2‐x_ from the TiO_2_ bulk to the metal particles. Overall, the Ti affinity of metals and the diffusion coefficient of metal in metal oxide, serve as reliable predictors for SMSI behavior and encapsulation kinetics.

## 4 Exploring the SMSI Terrain on TiO_2_: A Comprehensive Analysis of Critical Factors

### 4.1 Phase Matters: Crystalline Structures of TiO_2_ and SMSI

TiO_2_ exists in three main crystalline forms: anatase (TiO_2_‐A), rutile (TiO_2_‐R), and brookite (TiO_2_‐B). Among these, anatase and rutile are the most commonly utilized in heterogeneous catalysis. Each of these forms possesses unique physical and chemical properties, including differences in thermal stability, density, band gap, and surface structure. The crystal phase of TiO_2_ plays a crucial role in influencing the SMSI effect. An in situ electron paramagnetic resonance (EPR) spectroscopic study, using CO as a probe molecule, examined the SMSI behavior in Pd/TiO_2_‐A and Pd/TiO_2_‐R.^[^ ^68^ ^]^ The catalysts were reduced at two different temperatures under hydrogen: 200 °C and 450 °C. SMSI was detected on Pd/TiO_2_‐A when reduced at 200 °C, whereas no SMSI was observed for Pd/TiO_2_‐R under the same conditions. This difference is attributed to the fact that Ti^3+^ ions in TiO_2_‐R are more stable and remain fixed on the surface, as rutile TiO_2_ is more thermodynamically and structurally stable than anatase. The greater stability of the rutile phase inhibits significant diffusion of Ti^3+^ ions onto the Pd nanoparticles. Conversely, in the less stable anatase phase, Ti^3+^ ions are more prone to migrate to the surface of the Pd nanoparticles, thereby promoting the formation of SMSI. However, when the reduction temperature was increased to 450 °C, SMSI was observed in both catalysts. At this higher temperature, the diffusion of Ti^3+^ ions is enhanced, enabling them to overcome surface binding in both anatase and rutile TiO_2_.

**Figure 4:**
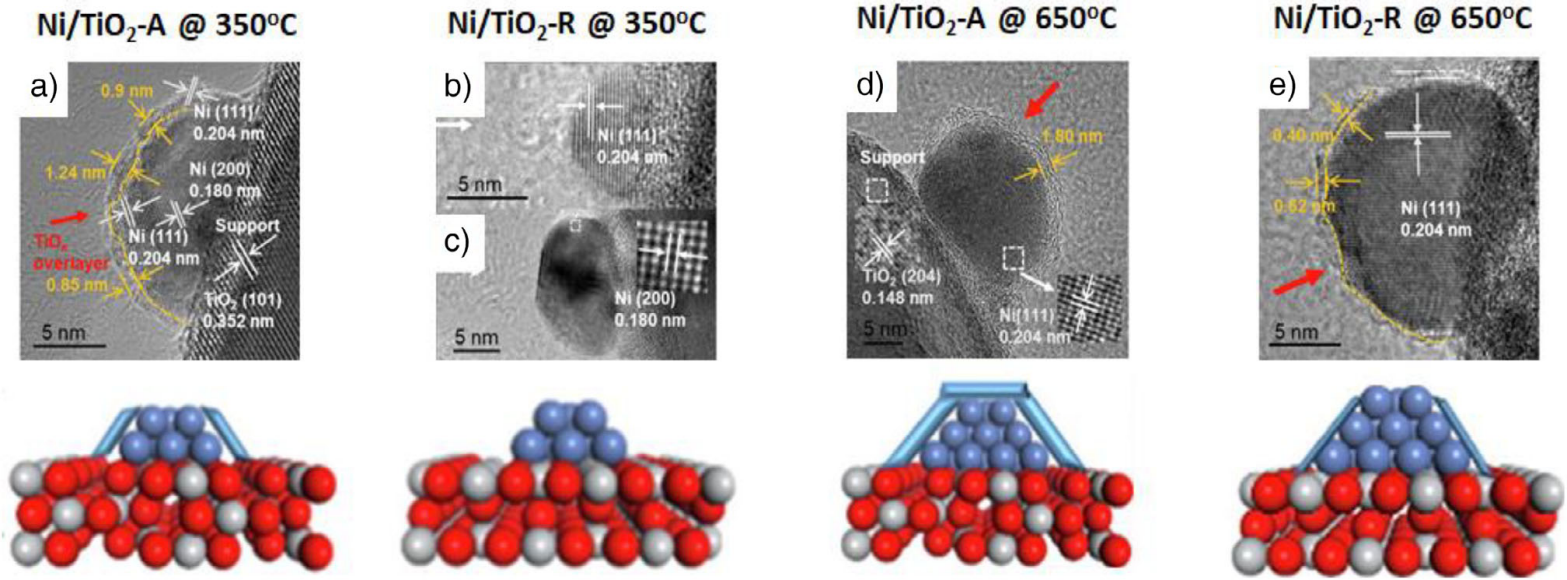
TEM images of Ni/TiO_2_‐A and Ni/TiO_2_‐R at different reduction temperatures showing different extent of encapsulation: a) Ni/TiO_2_‐A at 350 °C; b,c) Ni/TiO_2_‐R at 350 °C; d) Ni/TiO_2_‐A at 650 °C; e) Ni/TiO_2_‐R at 650 °C. Reprinted with permission from Ref. [69]. Copyright 2022, Elsevier.

A similar observation was noted with Ni/TiO_2_‐A and Ni/TiO_2_‐R catalysts.^[^ ^69^ ^]^ In the case of Ni/TiO_2_‐A, SMSI was observed when the catalyst was reduced at 350 °C under H_2_, leading to only partial encapsulation of Ni nanoparticles by TiO_2_‐A (Figure 4a). However, no SMSI was detected on Ni/TiO_2_‐R under the same conditions (Figure 4b,c). When the reduction temperature was increased to 650 °C, complete encapsulation of the Ni nanoparticles occurred on TiO_2_‐A, resulting in a thicker overlayer (Figure 4d). In contrast, this higher temperature induced only moderate SMSI on Ni/TiO_2_‐R (Figure 4e). The H_2_‐temperature programmed reduction (TPR) results showed that the H_2_/Ni molar ratio (1.30) for Ni/TiO_2_‐A was higher than the stoichiometric ratio, indicating greater reduction of TiO_2_‐A. On the other hand, the H_2_/Ni molar ratio for Ni/TiO_2_‐R was close to the theoretical ratio of 1.14, suggesting the very limited reduction of TiO_2_‐R.

A subsequent study also confirmed complete encapsulation of Ni particles for Ni/TiO_2_‐A upon reduction at 500 °C, whereas only partial encapsulation was observed for Ni/TiO_2_‐R.^[^ ^70^ ^]^ This difference was once again attributed to the distinct reduction behaviors of TiO_2_‐A and TiO_2_‐R, as further validated by H_2_‐TPR analysis: TiO_2_‐A readily reduced at ∼500 °C, whereas TiO_2_‐R required temperatures exceeding 700 °C. Extended X‐ray absorption fine structure (EXAFS) analysis provided additional insight, revealing a Ti‐O coordination number of 3.9 for Ni/TiO_2_‐A and 4.6 for Ni/TiO_2_‐R, both significantly lower than the reference TiO_2_ value of 6.0. This suggests that TiO_2_‐A facilitates stronger encapsulation of Ni particles, forming a defective TiO_2‐x_ overlayer. The lower surface Ni/Ti atomic ratio in Ni/TiO_2_‐A (0.14) compared to Ni/TiO_2_‐R (0.34) further supports a higher coverage of Ni by TiO_2_‐A. Moreover, the percentage of Ti^3+^ species was higher in Ni/TiO_2_‐A (18.5%) than in Ni/TiO_2_‐R (11.2%), with a similar trend observed for oxygen vacancies: 37.1% in Ni/TiO_2_‐A compared to 28.3% in Ni/TiO_2_‐R. These findings indicate that Ni/TiO_2_‐A undergoes greater oxygen vacancy formation during reduction. This is consistent with the high degree of Ni particles encapsulation (92.6%) observed for Ni/TiO_2_‐A, further reinforcing the stronger SMSI effect in the anatase‐supported catalyst.^[^ ^70^ ^]^

The crystalline phase of TiO_2_ affects SMSI formation differently in Ru‐based catalysts when a calcination step precedes reduction.^[^ ^71^ ^]^ In Ru/TiO_2_‐A and Ru/TiO_2_‐R catalysts, SMSI conditions (H_2_ reduction at 300 °C) were applied after calcination at 400 °C. Interestingly, without calcination, neither catalyst exhibited SMSI, and both showed similar catalytic activity in CO_2_ hydrogenation reaction. However, calcination before reduction led to distinct behaviors: Ru/TiO_2_‐A displayed a conventional SMSI effect, whereas Ru/TiO_2_‐R showed strong interfacial coupling without SMSI. This difference stems from RuO_2_ formation during calcination. RuO_2_, with a lattice misfit of less than 3.0% with TiO_2_‐R, forms epitaxial overlayers on TiO_2_‐R. These interfacial RuO_2_ species anchor Ru nanoparticles, creating flat particles with low contact angles and high curvature (metal–metal versus metal–oxide interactions). After H_2_ reduction, Ru on TiO_2_‐R partially retains its epitaxial structure, whereas on TiO_2_‐A, Ru exists only as nanoparticles. XPS analysis confirmed this, showing a Ru^δ+^/Ru° (oxidized/reduced) ratio of 2.2 for Ru/TiO_2_‐R and 0.2 for Ru/TiO_2_‐A. This is how the crystal phase of TiO_2_ influences SMSI when metals with similar lattice parameters are loaded over TiO_2_.^[^ ^71^ ^]^ A similar trend was observed in Rh nanoparticles supported on TiO_2_‐A and TiO_2_‐R catalysts, where the metal/TiO_2_‐A catalyst exhibited a higher SMSI than its rutile counterpart.^[^ ^69^, ^72^, ^73^, ^74^ ^]^

Another study on Pt/TiO_2_‐A, Pt/TiO_2_‐R, and Pt/TiO_2_‐P25 (which contains approximately 8 wt% rutile and remaining anatase, according to X‐ray diffraction (XRD) analysis) reduced under hydrogen at 500 °C confirmed that SMSI is significantly stronger in Pt/TiO_2_‐A compared to Pt/TiO_2_‐R.^[^ ^75^ ^]^ Additionally, the study revealed that SMSI in Pt/TiO_2_‐P25 is even more pronounced than in either Pt/TiO_2_‐A or Pt/TiO_2_‐R. Overall, these findings emphasize the significance of tailoring the crystalline phase and reduction conditions to achieve the desired SMSI characteristics, which are essential for optimizing catalytic performance.

### 4.2 The Facet Effect: The Influence of TiO_2_ Surface Orientation

**Figure 5:**
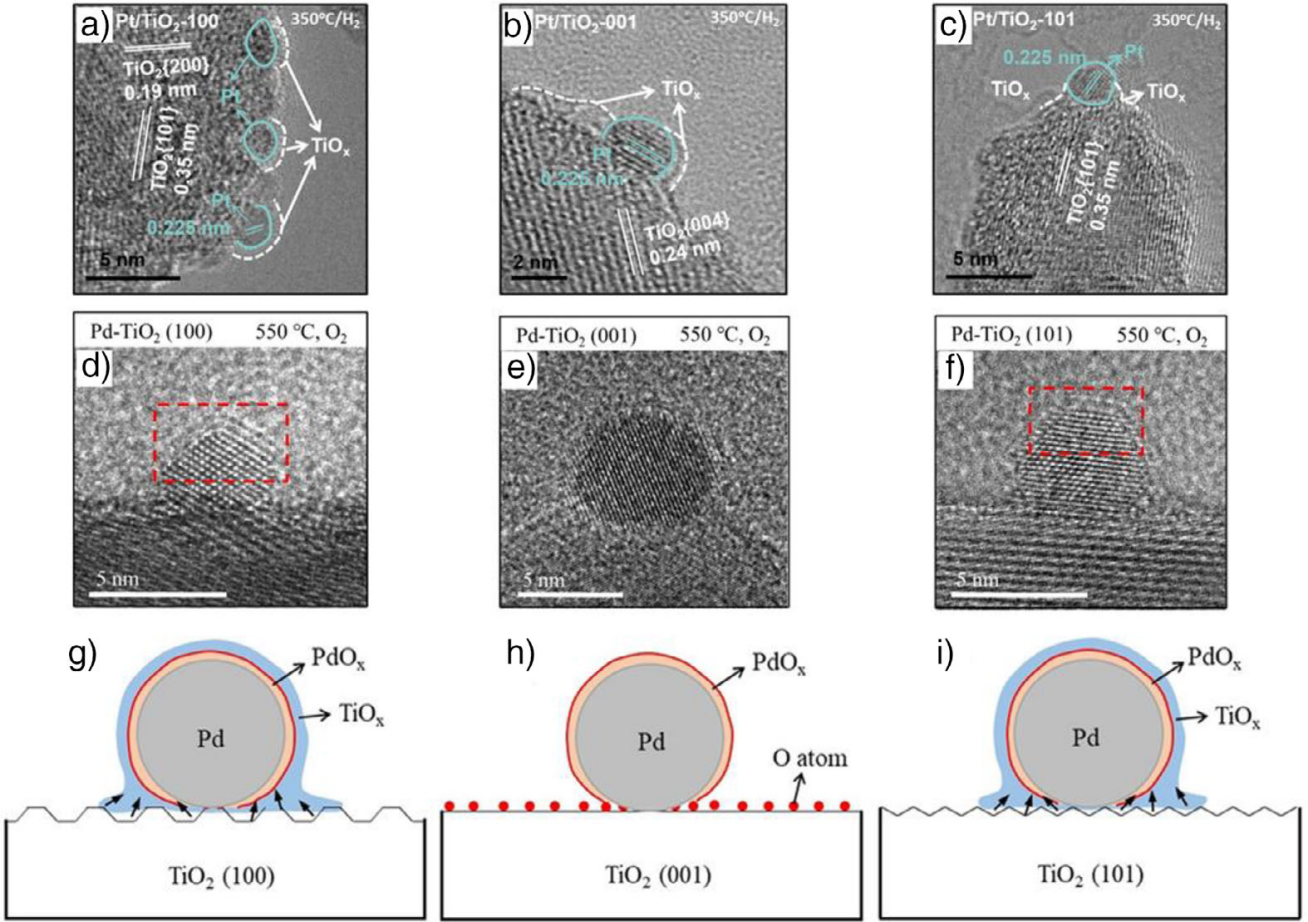
Facet dependent SMSI of Pt(Pd)/TiO_2_. a–c) TEM images of Pt/TiO_2_‐A with various exposed facets following H_2_ reduction at 350 °C for 1 h, revealing differing degrees of encapsulation. a) Pt/TiO_2_‐A(100), b) Pt/TiO_2_‐A(001), and c) Pt/TiO_2_‐A(101). Reprinted with permission from Ref. [76]. Copyright 2023, Elsevier. d–f) TEM images of Pd/TiO_2_‐A with various exposed facets following O_2_ treatment at 550 °C showing different degree of encapsulation: d) Pd/TiO_2_‐A(100), e) Pd/TiO_2_‐A(001), and f) Pd/TiO_2_‐A(101). g–i) Shows the representation of different degree of SMSI corresponding to (d–f). Reprinted with permission from Ref. [78]. Copyright 2021, WILEY‐VCH.

In general, the SMSI effect of TiO_2_ is highly dependent on the exposed facet and the gas environment during the temperature treatment. The encapsulation behavior of TiO_2_ is strongly influenced by the crystal planes it exposes, which can be altered by utilizing different TiO_2_ morphologies. A model study examined the encapsulation degree of Pt/TiO_2_‐A catalyst at 350 °C under hydrogen. The focus was on the (100), (101), and (001) crystal planes of TiO_2_‐A, which correspond to the rod, tetragonal bipyramid, and sheet morphologies, respectively.^[^ ^76^ ^]^ Complete encapsulation was observed for Pt/TiO_2_‐A(100) (Figure 5a), whereas partial encapsulation occurred for Pt/TiO_2_‐A(001) (Figure 5b). On Pt/TiO_2_‐A(101), the TiO_x_ layers only encapsulated the bottom of the Pt nanoparticles (Figure 5c). This was in accordance with the decrease in CO chemisorption and followed the order Pt/TiO_2_‐A(100) (65%) > Pt/TiO_2_‐A(001) (50%) > Pt/TiO_2_‐A(101) (33%), correlating with the degree of encapsulation. The variation in encapsulation among different TiO_2_ facets was associated with the formation of oxygen vacancies on each facet. TiO_2_‐A(100), TiO_2_‐A(001), and TiO_2_‐A(101) were estimated to reduce to a stoichiometry of TiO_1.961_, TiO_1.993_, and TiO_1.994_, respectively. Oxygen vacancies, measured by EPR spectroscopy, were found to be 1.412 × 10^12^, 5.018 × 10^11^, and 1.423 × 10^11^ spins mm^−3^ for Pt/TiO_2_‐A(100), Pt/TiO_2_‐A(001), and Pt/TiO_2_‐A(101), correspondingly. Moreover, on the (101) surface, the oxygen vacancy will migrate into the subsurface and bulk, restoring the oxygen content at the surface.^[^ ^77^ ^]^ These findings suggest that the extent of TiO_2_ encapsulation is directly related to the degree of oxygen vacancy formation on its facets, which is influenced by TiO_2_ morphology.

In addition to developing under a hydrogen atmosphere, SMSI can also form in situ when the materials are exposed to an oxygen atmosphere (O‐SMSI).^[^ ^45^ ^]^ Similarly, the SMSI effect under an oxygen atmosphere on Pd/TiO_2_‐A with (100), (101), and (001) facets has also been investigated.^[^ ^78^ ^]^ An in situ TEM study showed that no encapsulation of Pd nanoparticles occurred on any of the catalysts when treated at 350 °C. However, at 550 °C, significant encapsulation was observed on Pd nanoparticles located on the TiO_2_‐A(100) (Figure 5d) and TiO_2_‐A(101) surfaces (Figure 5f). No encapsulation was detected on nanoparticles situated on the TiO_2_‐A(001) plane (Figure 5e). This pattern contrasts with the behavior observed under hydrogen atmosphere as discussed before.^[^ ^76^ ^]^ For the Pd/TiO_2_‐A(101) catalyst in an oxygen atmosphere, lattice spacing measurements revealed that the outermost single layer consists of TiO_x_, with a single layer of PdO_x_ directly beneath it (Figure 5i). In the case of the Pd/TiO_2_‐A(100) catalyst, two layers of TiO_x_ followed by two layers of PdO_x_ were observed (Figure 5g). For the Pd‐TiO_2_‐A(001) catalyst, only PdO_x_ layer was observed (Figure 5h).

### 4.3 The Heat Factor: The Role of Reduction and Calcination Temperatures in TiO_2_ Reduction and SMSI Encapsulation

Since SMSI on TiO_2_ is often observed at relatively low temperatures of 250 °C (Figure 1),^[^ ^63^ ^]^ understanding how reduction temperature influences the encapsulation degree of SMSI is crucial. A study on a Pt/TiO_2_‐P25 catalyst (P25 with 80% anatase and 20% rutile phase) examined this effect.^[^ ^79^ ^]^ The catalyst was reduced under hydrogen at 250, 350, and 550 °C. While bulk TiO_2_ typically reduces at temperatures above 500 °C without metal, the presence of Pt lowers this threshold. The average particle size of Pt remained relatively constant between 1.6–1.7 nm, regardless of whether it was reduced at 250 °C or 550 °C. However, CO chemisorption studies that account for the exposure of Pt, revealed notable differences at different reduction temperatures. This difference points the formation of SMSI. Accordingly, Pt dispersion was found to be 69% (250 °C) > 57% (350 °C) > 13.1% (550 °C) and SMSI intensifies with higher reduction temperatures. Besides, XPS analysis revealed that the Pt/Ti ratio decreased as the reduction temperature increased: 0.022 (250 °C) > 0.019 (350 °C) > 0.017 (550 °C) indicating that Pt becomes more deeply embedded in the SMSI layer of TiO_x_ at higher reduction temperatures. Correspondingly, the Ti^3+^/Ti^4+^ ratio increased, following the trend 0.036 (250 °C) < 0.113 (350 °C) < 0.247 (550 °C), confirming the enhanced partial reduction of TiO_2_ with rising reduction temperatures.

**Figure 6:**
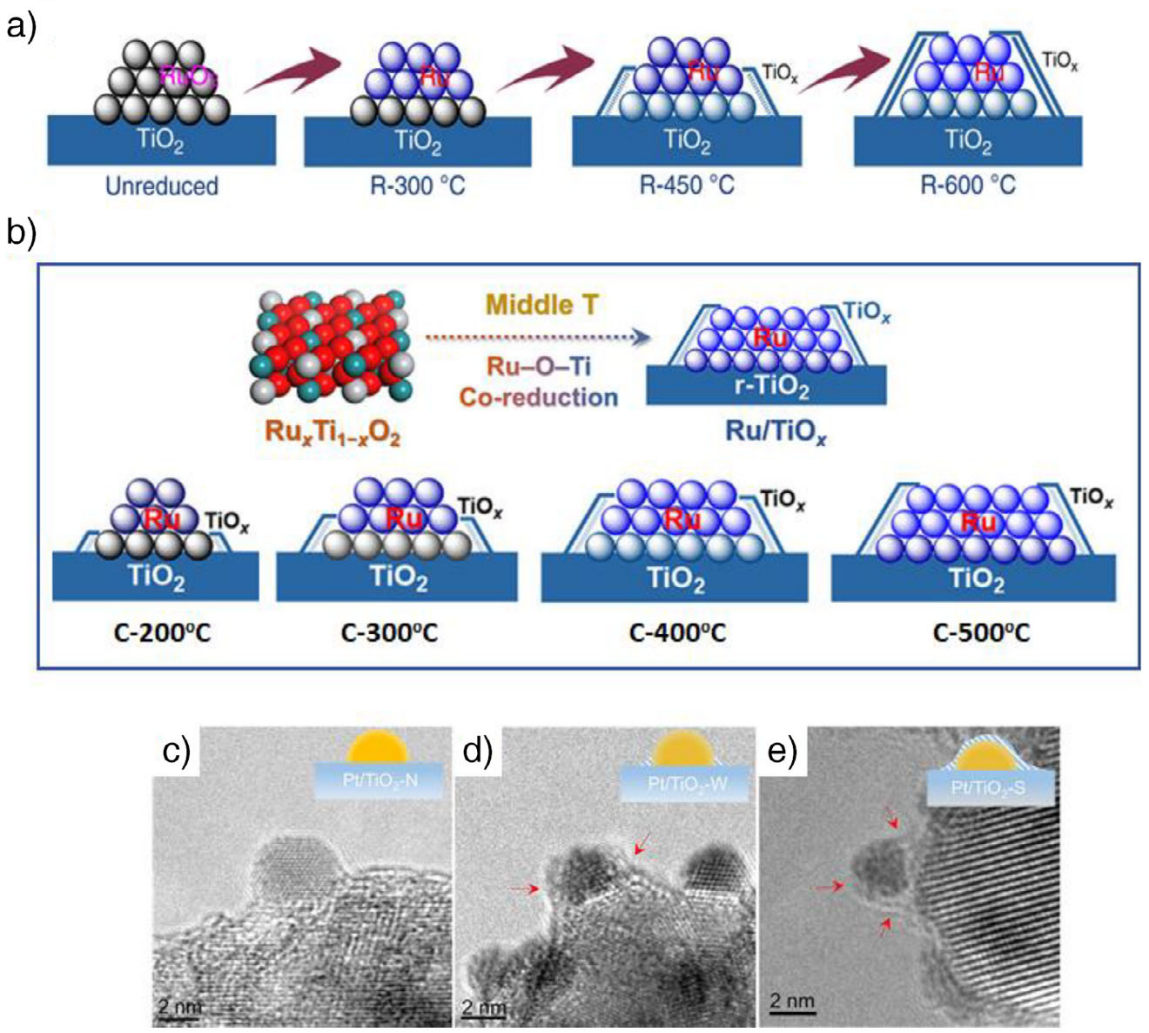
Effect of reduction and calcination temperatures on the extent of SMSI. a) Effect of reduction temperature of Ru/TiO_2_‐R on its SMSI. Reprinted with permission from Ref. [80]. Copyright 2020, Springer Nature. b) Effect of calcination temperature on the extent of SMSI on Ru/TiO_2_‐R catalyst. Reprinted with permission from Ref. [81]. Copyright 2022, American Chemical Society. c,d) Effect of the combination of calcination temperature and reduction temperature on the extent of SMSI on Pt/TiO_2_‐A. c) Calcination at 300 °C and reduction at 200 °C, “N” denotes no encapsulation. d) Calcination at 300 °C and reduction at 450 °C, “W” denotes weak encapsulation. e) Calcination at 500 °C and reduction at 450 °C, “S” denotes strong encapsulation. Reprinted with permission from Ref. [82]. Copyright 2023, American Chemical Society.

Moreover, the role of reduction temperature in controlling SMSI on a rutile TiO_2_ was explored using a Ru/TiO_2_‐R catalyst.^[^ ^80^ ^]^ The catalyst underwent reduction at 300 °C, 450 °C, and 600 °C under H_2_. Regardless of the reduction temperatures, the Ru particle sizes remained stable around 2 nm. The CO chemisorption values, however, showed a steady decrease in dispersion with higher reduction temperatures: 43.3% at 300 °C, 34.0% at 450 °C, and 17.5% at 600 °C indicating the different extent of SMSI. Based on high‐angle annular dark‐field scanning transmission electron microscopy (HAADF‐STEM) and EXAFS analyses, no evidence of SMSI was found in the catalyst reduced at 300 °C (Figure 6a), whereas a moderate level of SMSI appeared at 450 °C. At 600 °C, significant coverage of Ru nanoparticles by TiO_x_ indicated strong SMSI (Figure 6a). Several studies mentioned here (in previous sections–Figures 1 and 3, 4, 5 and in the sections that follow) also confirm the positive influence of reduction temperature in enhancing the SMSI effect on both anatase and rutile TiO_2_. These findings clearly demonstrate that higher reduction temperatures intensify the SMSI effect, resulting in increased partial reduction of TiO_2_ and greater encapsulation of metals by TiO_x_. Therefore, the reduction temperature is a key parameter in inducing and controlling the extent of SMSI.

Not only reduction temperature, the calcination temperature also plays a significant role in influencing the extent of SMSI. This was evident in the behavior of a Ru/TiO_2_‐R catalyst, where both rutile TiO_2_ and RuO_2_ share the same crystal structure and have similar lattice parameters.^[^ ^81^ ^]^ On the other hand, the lattice types of anatase TiO_2_ and RuO_2_ are different.^[^ ^71^ ^]^ Calcination of Ru/TiO_2_‐R promotes the formation of a chemical solid solution between RuO_2_ and TiO_2_‐R, leading to the formation of the Ru_x_Ti_1–x_O_2_ phase. Higher calcination temperatures also promote epitaxial growth of RuO_2_ on the TiO_2_‐R, further enhancing the formation of this phase. As the calcination temperature increases from 300 °C to 500 °C, the coordination number of Ru─O─Ti bonds increases from 0.7 to 0.9, indicating a more developed and pronounced Ru_x_Ti_1–x_O_2_ interphase at elevated temperatures. When the Ru/TiO_2_‐R catalyst was calcined at various temperatures (200, 300, 400, and 500 °C) and subsequently reduced at 450 °C in H_2_, varying degrees of SMSI were observed (Figure 6b). Notably, TiO_x_ overlayer encapsulation on Ru nanoparticles only occurred at calcination temperatures ≥ 300 °C. The presence of Ru─O─Ti bonds in the Ru_x_Ti_1–x_O_2_ interphase facilitated the co‐reduction of Ru and Ti during H_2_ treatment, enhancing the TiO_x_ overlayer formation on Ru nanoparticles (Figure 6b). Since the extent of the Ru_x_Ti_1–x_O_2_ interphase increases with higher calcination temperatures, the degree of SMSI correspondingly increased after the reduction.

Efforts were also made to exert greater control over the extent of SMSI by adjusting both the calcination and reduction temperatures. In the case of a Pt/TiO_2_‐A catalyst, varying these temperatures influenced the degree/extent of encapsulation.^[^ ^82^ ^]^ When the catalyst was calcined at 300 °C and reduced at 200 °C, no encapsulation occurred (Figure 6c). However, raising the reduction temperature to 450 °C resulted in partial encapsulation (Figure 6d). The highest level of encapsulation was observed when the catalyst was calcined at 500 °C and reduced at 450 °C (Figure 6e). Thus, higher calcination and reduction temperatures promote greater encapsulation. These studies demonstrate that the degree of encapsulation can be controlled by either increasing the reduction temperature while keeping the calcination temperature constant or by adjusting the calcination temperature while maintaining the reduction temperature.

### 4.4 Scaling Down: SMSI's Sensitivity to TiO_2_ Particle Size

The particle size of TiO_2_ plays a crucial role in determining the extent of SMSI, making it a significant variable to consider in this context. The effect of TiO_2_ particle size on the SMSI in a Pd/TiO_2_‐A catalyst was reported using TiO_2_ particles of 0.1 µm and 14 nm.^[^ ^83^ ^]^ When reduced at 500 °C, SMSI was detected in the Pd/TiO_2_‐A‐14 nm catalyst, whereas it was absent in the Pd/TiO_2_‐A‐0.1 µm catalyst. The presence of SMSI in the 14 nm catalyst maintained the Pd particle size (increased stability), whereas, in the 0.1 µm catalyst, high‐temperature reduction led to an increase in Pd particle size due to sintering. The TiO_2_‐A‐14 nm catalyst exhibited a surface Ti/O ratio that was approximately 1.1 times higher than that of the TiO_2_‐A‐0.1 µm catalyst, indicating a greater number of oxygen vacancies in the smaller particles, which induces SMSI formation. EPR studies further supported these findings by showing a higher concentration of surface Ti^3+^ in the TiO_2_‐A‐14 nm catalyst. In contrast, the TiO_2_‐A‐0.1 µm catalyst had more Ti^3+^ in the bulk than on the surface. Since surface Ti^3+^ can easily migrate to Pd nanoparticles, SMSI was observed in the TiO_2_‐A‐14 nm catalyst but not in the TiO_2_‐A‐0.1 µm catalyst after reduction. This is consistent with findings from other studies too,^[^ ^84^ ^]^ where smaller TiO_2_ particles were found to be more easily reducible than larger ones in H_2_‐TPR studies with Pd/TiO_2_ catalysts.

In another study, the impact of TiO_2_ particle size on SMSI (Pt/TiO_2_‐A) was examined using particles of < 4, 4.4, and 7.5 nm.^[^ ^85^ ^]^ The results consistently showed that the SMSI effect weakened as the TiO_2_ particle size increased. Since the reduction of TiO_2_ is a crucial step for SMSI, smaller TiO_2_ particles are expected to exhibit SMSI at lower reduction temperatures than larger particles. Additionally, most studies in the literature use TiO_2_ in the nanometer range, where SMSI is typically observed at or below an average reduction temperature of 500 °C. These findings suggest that optimizing the particle size of TiO_2_ is another way to achieve effective SMSI with TiO_2_ supports, with smaller TiO_2_ nanoparticles being more favorable for SMSI formation at standard reduction temperatures.

## 5 Engineering SMSI: Innovative and Nonclassical Approaches for Controlled SMSI

In addition to the previously mentioned approaches that influence and enable modulation of SMSI on TiO_2_‐based systems, various chemical methods can be employed to further regulate SMSI, maximizing metal exposure while preserving its beneficial effects. These approaches fall under the category of nonclassical SMSI, where the TiO_2_ support is typically chemically modified prior to metal deposition and SMSI induction.

### 5.1 Alkali and Alkaline Metals: New Additives for Optimizing SMSI

**Figure 7:**
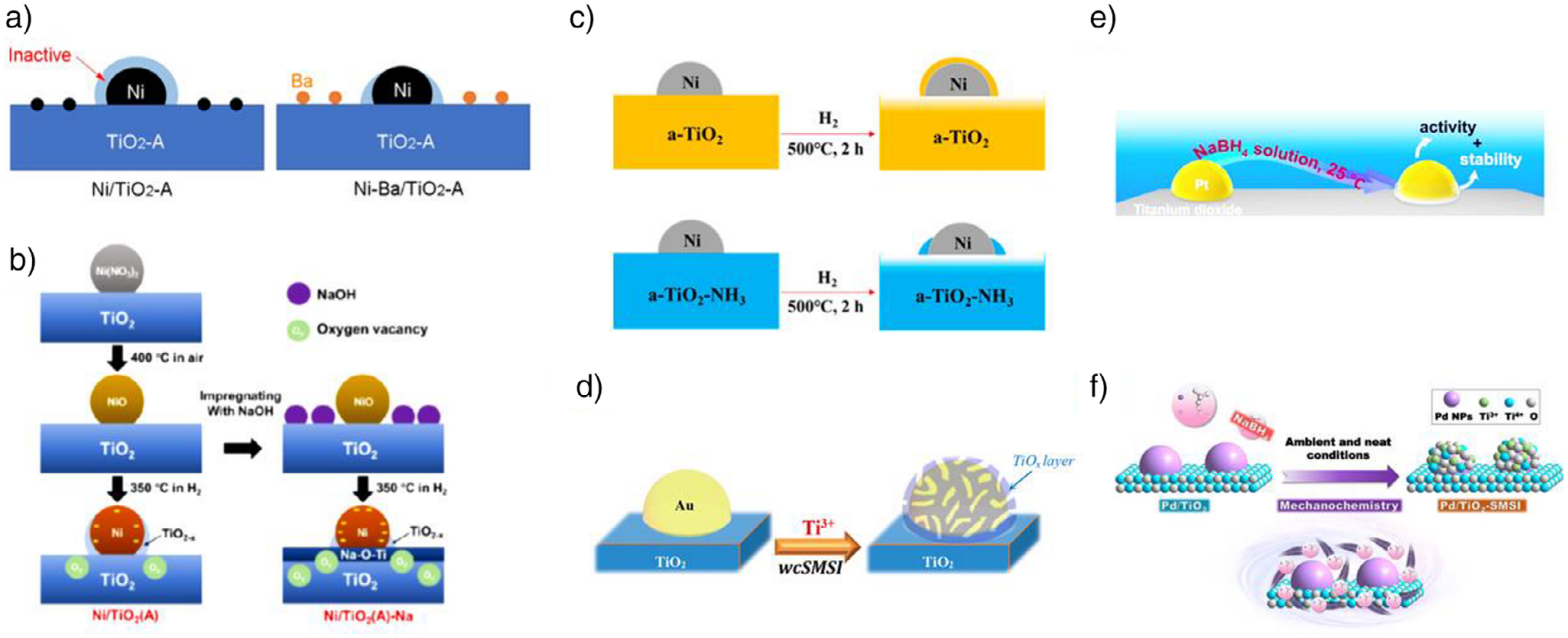
Different chemical methods to regulate SMSI on metal/TiO_2_. a) Effect of Ba additive on SMSI of Ni/TiO_2_‐A catalyst, Reprinted with permission from Ref. [86]. Copyright 2024, American Chemical Society. b) Effect of NaOH treatment on the SMSI of Ni/TiO_2_‐A. Reprinted with permission from Ref. [87]. Copyright 2021, American Chemical Society. c) Effect of NH_3_ treatment of TiO_2_ on SMSI, Reprinted with permission from Ref. [88]. Copyright 2019, American Chemical Society. d) Wet‐chemistry: deposition of Ti^3+^ colloidal solution on Au/TiO_2_ for regulating SMSI._,_ Reprinted with permission from Ref. [33]. Copyright 2019, American Chemical Society. e) NaBH_4_ reduction approach to control SMSI on Pt/TiO_2_. Reprinted with permission from Ref. [21]. Copyright 2024, American Chemical Society. f) Mechanochemical treatment with NaBH_4_ and its influence on SMSI in Pd/TiO_2_. Reprinted with permission from Ref. [90]. Copyright 2023 American Chemical Society.

Modifying TiO_2_ with other elements is a commonly employed method to control SMSI. For instance, in barium (Ba)‐modified TiO_2_ catalyst like Ni‐Ba/TiO_2_‐A, the SMSI effect was more effectively managed than in the unmodified Ni/TiO_2_‐A, where full encapsulation occurred when reduced at 400 °C under H_2_ (Figure 7a).^[^ ^86^ ^]^ As the Ba loading increased, the catalyst increasingly inhibited SMSI, thus regulating it effectively. Even a small amount of Ba (0.005–0.04 mmol g^−1^) was sufficient to control the SMSI, representing only about 0.9%–7.3% monolayer coverage of Ba atoms on the TiO_2_‐A surface. HAADF‐STEM analysis showed that Ba was evenly distributed at the atomic level on Ni‐Ba/TiO_2_‐A, which maximized the interaction between Ba and TiO_2_‐A. This stronger interaction with increasing Ba loading was responsible for the effective inhibition of SMSI. According to DFT calculations, the most stable bonding of Ba on the TiO_2_ (101) surface occurs when Ba is bonded to two bridging oxygen atoms. High‐temperature reduction of TiO_2_‐A under hydrogen leads to the formation of oxygen vacancies by removing these bridging oxygen atoms. However, when Ba bonds with these two bridging oxygens, it stabilizes the Ti–O–Ti structures, making them less prone to reduction and thereby diminishing the SMSI effect. The fact that SMSI is reduced even at lower Ba loadings, where there are fewer Ba─O─Ti bonds and more reducible Ti─O─Ti bonds, suggests that Ba not only prevents the reduction and movement of the Ti–O structure it bonds to, but also acts as a “blocker” that hinders the migration of nearby Ti and O atoms in the TiO_2_ lattice to Ni. Yet, the inhibitory effect of Ba depends on the reduction temperature employed. Higher temperatures above 400 °C encourage the diffusion of reduced Ti species onto Ni particles. Nevertheless, higher Ba loadings can still provide better control over encapsulation even at higher reduction temperatures of 500 °C. However, complete encapsulation was observed at a higher reduction temperature (600 °C). In brief, modifying TiO_2_ with barium effectively controls the SMSI effect by stabilizing Ti–O structures and reduces encapsulation, with the degree of control dependent on both Ba loading and reduction temperature.

Similarly, to barium, sodium (Na) modification of TiO_2_‐A offers another method to regulate SMSI. In the case of Na‐modified TiO_2_‐A, the Ni/TiO_2_‐A‐Na catalyst effectively showcased this control.^[^ ^87^ ^]^ After reductive treatment at 350 °C, almost complete encapsulation of Ni particles was observed in the unmodified Ni/TiO_2_‐A catalyst. However, in the Na‐modified Ni/TiO_2_‐A‐Na catalyst, only partial encapsulation occurred (Figure 7b). This difference was attributed to the formation of a thermodynamically stable Na_2_TiO_3_ phase on the surface of TiO_2_‐A, which has a lower capacity for encapsulation compared to TiO_2_‐A. Just as Ba modification regulates SMSI by stabilizing the Ti‐O‐Ti structures and lessening the encapsulation, Na modification achieves a similar outcome, however through a different mechanism by forming a phase that inherently weakens encapsulation tendency. Therefore, Na modification provides an alternative strategy to control the SMSI behavior, highlighting the versatility of alkali and alkaline earth metals modification in controlling this interaction.

### 5.2 Controlling SMSI Through NH_3_ Treatment of TiO_2_

To modulate the SMSI by reducing excess oxygen vacancy formation, an NH_3_ treatment of TiO_2_ acts as an additional strategy. For example, treating a Ni/TiO_2_‐A catalyst, where the TiO_2_‐A was pretreated with NH_3_, provides an effective approach to controlling SMSI.^[^ ^88^ ^]^ At a reduction temperature of 500 °C, complete encapsulation of Ni nanoparticles occurs on Ni/TiO_2_‐A, which can obstruct the majority of the active catalytic sites. To counteract this, the TiO_2_‐A support was treated with NH_3_ at 600 °C. After loading Ni and reducing at 500 °C, only partial encapsulation of the Ni nanoparticles was observed (Figure 7c), allowing for greater exposure of Ni sites. EPR analysis revealed that NH_3_ treatment induced the formation of Ti^3+^ in the TiO_2_‐A‐NH_3_ support, with the amount of Ti^3+^ increasing with the duration of NH_3_ exposure. Notably, no Ti^3+^ was detected in untreated TiO_2_‐A. Interestingly, the oxygen vacancies, typically important for SMSI formation, were absent in the NH_3_‐treated sample. This substantial presence of Ti^3+^ and reduced or absence of oxygen vacancies likely explains the reduced SMSI effect in NH_3_‐treated catalyst. While NH_3_ treatment represents an additional approach to controlling SMSI, its efficacy remains debatable, as hydrogen treatment along with careful control of calcination and reduction temperatures allows achieving similar results. However, NH_3_ treatment becomes particularly advantageous when a shift in reaction selectivity is desired. For instance, NH_3_ treatment in a Ni/TiO_2_‐A‐NH_3_ catalyst led to a shift in selectivity from CO to CH_4_ as product of CO_2_ hydrogenation.^[^ ^88^ ^]^

### 5.3 Employing Wet‐Chemical Methods and Alternative Reducing Agents for Controlled SMSI

To prevent metal nanoparticles from growing during high‐temperature reduction‐especially with metals like Au, which has a low Tammann temperature (395 °C) a wet chemical method was used to generate a controlled SMSI on Au/TiO_2_‐A.^[^ ^33^ ^]^ This process involved preparing a TiO_x_ colloidal solution from TiCl_3_ and mixing it with a pre‐reduced, size‐controlled Au colloidal solution, followed by adding the TiO_2_‐A support. After heating at 300 °C, the catalyst turned into a porous TiO_x_‐encapsulated Au/TiO_2_‐A structure (Figure 7d). This approach can also be applied to other noble metals like Pt, Pd, and Rh, resulting in the formation of stable TiO_x_‐coated catalysts while preserving their size.

The use of NaBH_4_ as a reducing agent can modulate SMSI, as demonstrated for Pt/TiO_2_ (Figure 7e).^[^ ^21^ ^]^ In the related study, the Pt/TiO_2_ catalyst (which contains both anatase and rutile phases) was initially reduced at 200 °C under H_2_, followed by treatment with NaBH_4_ at room temperature to reduce Ti^4+^ to Ti^3+^. The CO chemisorption analysis revealed a marked decrease in CO uptake, from 25.4 µmol g^−1^ for the hydrogen‐reduced catalyst to 5.2 µmol g^−1^ after NaBH_4_ treatment. HRTEM analysis of the H_2_‐reduced catalyst displayed uncovered Pt particles, whereas after NaBH_4_ treatment, both exposed Pt nanoparticles and Pt nanoparticles partially encapsulated by a nonuniform TiO_x_ layer were observed. This indicates the occurrence of SMSI, albeit to a lesser extent. The absence of high‐temperature treatment limited TiO_x_ formation and migration, resulting in a lower degree of encapsulation compared to conventional SMSI. Other reducing agents like hydrazine^[^ ^21^ ^]^ and formaldehyde (HCHO)^[^ ^89^ ^]^ can also effectively achieve controlled SMSI in metal/TiO_2_ systems under milder conditions.

Moreover, SMSI has been successfully achieved for Pd/TiO_2_‐A through mechanochemical reduction with NaBH_4_ (Figure 7f),^[^ ^90^ ^]^ showing the method's versatility across various metals and synthesis techniques. These advanced methods, encompassing both wet chemical methods, alternative low temperature reducing agents, and mechanochemistry provide flexible solutions for stabilizing metal nanoparticle sizes and achieving controlled SMSI in a range of noble metals and TiO_2_. However, the environmental sustainability of these methods continues to pose significant challenges, highlighting the need for more eco‐friendly approaches.

## 6 In Situ Generation of SMSI During Catalysis: The Role of Carbonaceous Species from Reactants

**Figure 8:**
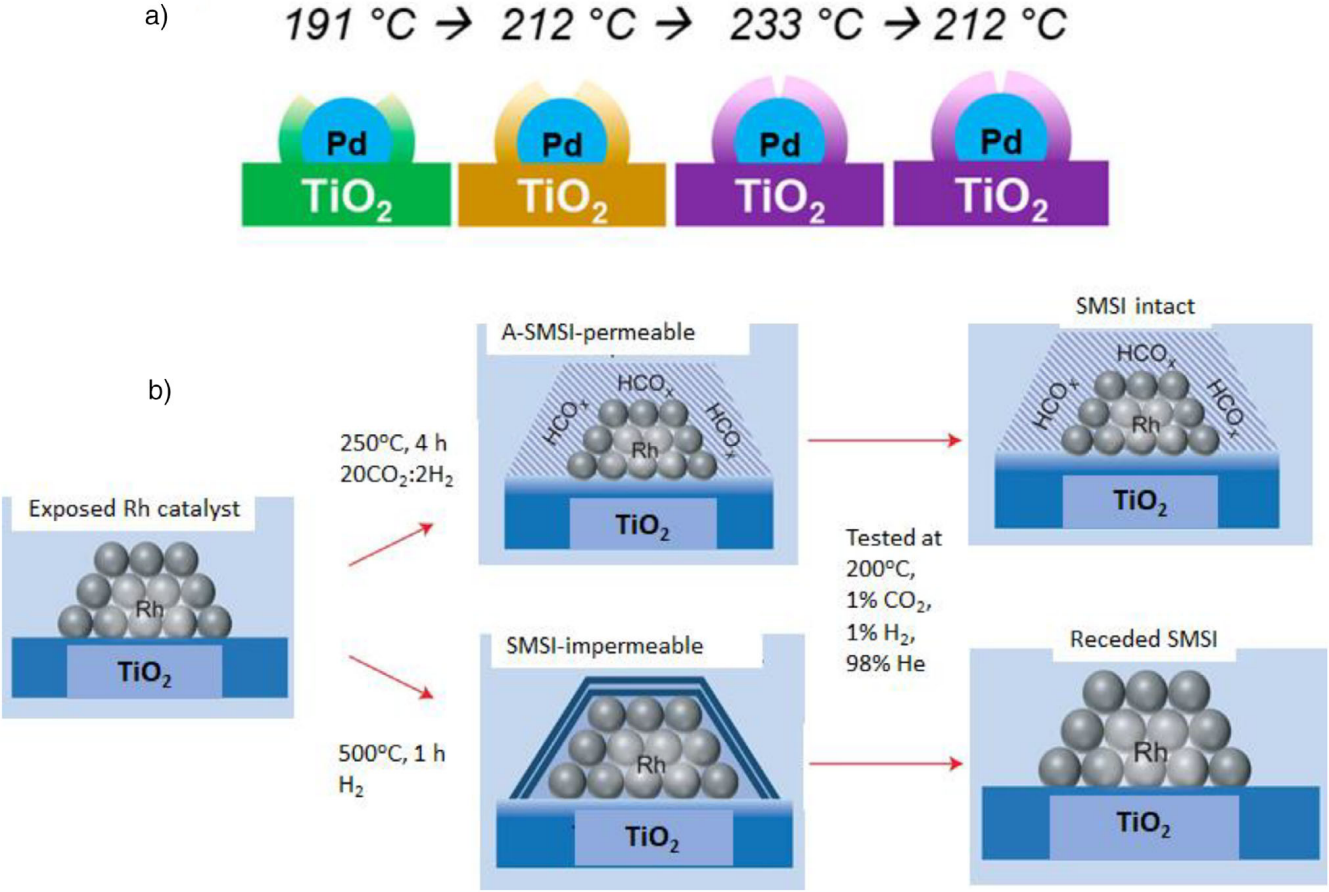
In situ formation of SMSI under reaction conditions. a) SMSI formation extent on Pd/TiO_2_‐A as temperature increases during 2‐propanol dehydration. Reprinted with permission from Ref. [91]. Copyright 2021, American Chemical Society. b) Adsorbate‐induced SMSI on Rh/TiO_2_‐P25 under a 20CO_2_:2H_2_ gas mixture, with stability compared to C‐SMSI. Reprinted with permission from Ref. [22]. Copyright 2017, Springer Nature.

The in situ formation of SMSI takes place under specific reaction conditions during catalysis. This process commonly involves reactive gases such as H_2_, O_2_, CO_2_, and various reactants. For example, during the dehydrogenation of 2‐propanol to acetone and H_2_, SMSI began to develop on a Pd/TiO_2_‐A catalyst in situ at a reaction temperature as low as 100 °C.^[^ ^91^ ^]^ The encapsulation layer that formed was highly unstable and retracted when the catalyst was exposed to O_2_ at room temperature. However, the retraction kinetics were slow, requiring nearly 15 h to complete. The evolution of this in situ SMSI was tracked using a temperature cycle. As the temperature increased from 190 °C to 212 °C, the extent of encapsulation of Pd nanoparticles increased (Figure 8a). A further rise in temperature to 233 °C led to an even greater SMSI effect. However, when the temperature was lowered back to 212 °C, the SMSI did not reverse as it had during the initial heating phase; instead, it remained as pronounced as it was at 233 °C. DFT calculations explored whether the presence of carbon contributes to the formation of the TiO_x_ overlayer at low temperatures. A Ti_2_O_3_ monolayer on the Pd surface indicated that carbon strongly adsorbs in the sublayers of the Pd nanoparticles, beneath the Ti_2_O_3_ layer. The SMSI induced by 2‐propanol is facilitated by carbonaceous species adsorbed at the interface between the Pd nanoparticles and the TiO_x_ overlayer, leading to an adsorbate‐induced SMSI. These results emphasize the temperature‐dependent nature of in situ SMSI and the important role of surface species from reactants in influencing the interaction.

A similar adsorbate‐induced SMSI was observed on a Rh/TiO_2_‐P25 catalyst during CO_2_ hydrogenation, even at the relatively low temperature of 250 °C with a 20CO_2_:2H_2_ gas mixture.^[^ ^22^ ^]^ In comparison, C‐SMSI on the same catalyst occurred at a higher temperature of 500 °C. In situ diffuse‐reflectance infrared Fourier transform spectroscopy (DRIFTS) studies of Rh/TiO_2_‐P25 under the 20CO_2_:2H_2_ mixture revealed significantly increased coverage of formate (HCO_2_) and bicarbonate (HCO_3_) species on the TiO_2_‐P25 surface. This enhanced presence of HCO_x_ species led to the formation of oxygen vacancies on TiO_2_, promoting its reduction and subsequent migration of Ti onto the Rh particles. In situ STEM analysis further showed the development of an amorphous layer over the Rh nanoparticles, containing Ti in a mixed state (30% Ti^3+^ and 70% Ti^4+^). In contrast, the C‐SMSI displayed a crystalline overlayer with Ti exclusively in the Ti^3+^ state. Additionally, the C‐SMSI gradually vanished during the reaction, whereas the HCO_x_‐induced SMSI, remained stable, allowing reactants to diffuse through (porous) (Figure 8b). The results further emphasize that an adsorbate‐induced SMSI can occur at significantly lower temperatures than classical SMSI, with the former exhibiting greater stability under reaction conditions, thereby enhancing catalyst performance by facilitating reactant diffusion.

The in situ SMSI is also dependent on the crystal phase of TiO_2_. On Rh/TiO_2_ with TiO_2_‐A, TiO_2_‐R, and TiO_2_‐P25, the SMSI state was induced in situ under CO_2_ hydrogenation conditions (24 vol% CO_2_ + 72 vol% H_2_ balanced with Ar).^[^ ^92^ ^]^ On Rh/TiO_2_‐A, the SMSI state emerged at 250 °C, whereas Rh/TiO_2_‐R exhibited only weak SMSI, which occurred at a higher temperature of 400 °C. This difference is attributed to the greater reducibility of TiO_2_‐A compared to TiO_2_‐R. In contrast, Rh/TiO_2_‐P25 did not exhibit SMSI even at 400 °C. These findings emphasize that the TiO_2_ crystal phase plays a crucial role in determining the temperature at which SMSI is activated under CO_2_ hydrogenation conditions.^[^ ^92^ ^]^

These insights demonstrate that the formation and evolution of SMSI are highly dependent on the presence of surface species derived from reactants, the reaction temperature, and the crystalline phase of TiO_2_. Adsorbate‐induced SMSI, which can occur at lower temperatures than classical SMSI, exhibits enhanced stability and improved catalytic performance, making it an essential consideration in the design of advanced catalytic systems.

## 7 From Static Perceptions to Dynamic Paradigms: Exploring Pre‐SMSI Benefits and SMSI Dynamics During Catalysis

Numerous studies have demonstrated the existence of SMSI in metal/TiO_2_ systems before catalytic reactions, utilizing a combination of complementary characterization techniques as discussed in earlier sections. However, a critical question remains largely unaddressed in the literature: does this pre‐SMSI state remain static during catalysis, or does it evolve dynamically in response to reaction conditions? This gap in understanding is important, as the pre‐SMSI state has shown substantial advantages for catalytic performance, often leading to increased activity, altered selectivity, and enhanced stability‐factors that are crucial in catalysis research. To investigate this further, the following section will highlight first the benefits of the pre‐SMSI state on the performance of metal/TiO_2_ catalysts through illustrative examples. Building on this foundation, it will subsequently analyze studies that investigate SMSI under in situ and operando conditions to illuminate its dynamic characteristics. This dual focus will enhance our understanding of SMSI both before and during catalysis, revealing its true nature and implications for catalytic design and application.

### 7.1 Benefits of Pre‐SMSI States on Metal/TiO_2_ Catalysts: Activity, Selectivity, and Stability

**Figure 9:**
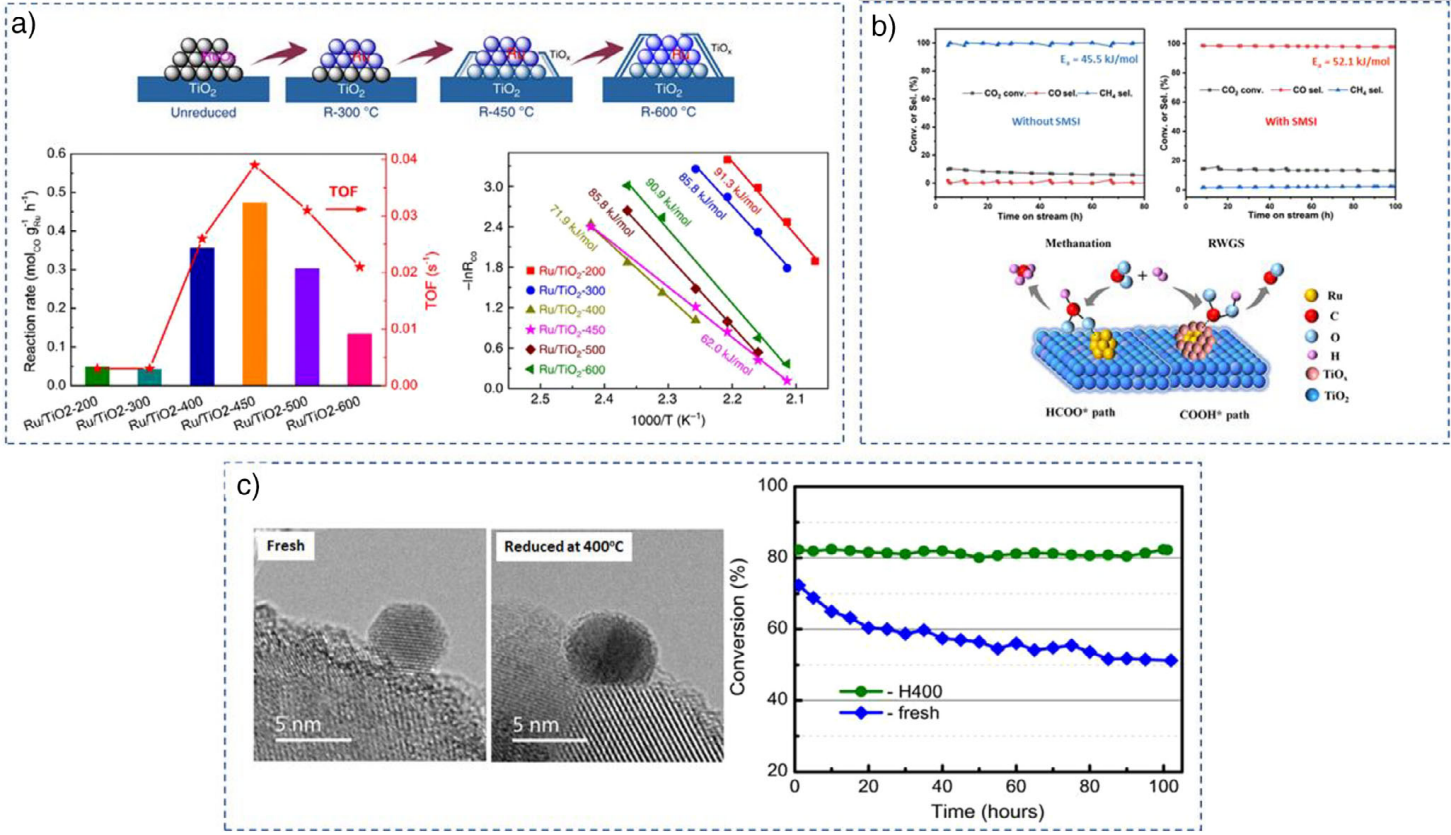
a) SMSI enhances the intrinsic activity of Ru/TiO_2_‐R in FTS, peaking at a reduction temperature of 450 °C, by lowering the activation energy. Reprinted with permission from Ref. [80]. Copyright 2020, Springer Nature. b) SMSI alters the selectivity of the Ru/TiO_2_‐A catalyst in CO_2_ hydrogenation over time, shifting from CH_4_ to CO due to changes in surface reaction intermediates. Reprinted with permission from Ref. [93]. Copyright 2024, American Chemical Society. c) SMSI enhances the stability of the Au/TiO_2_ catalyst (H400) during CO oxidation at 300 °C. The fresh catalyst shows no encapsulation, whereas reduction at 400 °C forms a noncrystalline, gas‐permeable overlayer of reduced TiO_2_ on the Au nanoparticles. Reprinted with permission from Ref. [32]. Copyright 2017, American Association for the Advancement of Science.

The enhancement of catalytic activity through the strategic design of metal‐support interfaces is a key focus in modern catalysis, exemplified by SMSI. This interaction facilitates the formation of new interfaces between the metal and the support, which serve as active sites and enhance catalytic activity. This phenomenon was observed in Ru/TiO_2_‐R catalysts during Fischer–Tropsch synthesis (FTS).^[^ ^80^ ^]^ SMSI started to form at reduction temperatures above 300 °C, accompanied by a decrease in Ru dispersion as the reduction temperature increased: 47.2% at 200 °C, 43.3% at 300 °C, 34% at 450 °C, and 17.5% at 600 °C. The intrinsic catalytic rate displayed a volcano‐like trend related to the extent of SMSI, reaching its maximum activity at 450 °C, which corresponded to the lowest apparent activation energy (E_a_) (Figure 9a). Mechanistically, the Ru/TiO_2_ interface plays a crucial role, with both metallic Ru and the TiO_x_ overlayer contributing to the overall activity. In the Ru/TiO_2_‐450 configuration, the optimized TiO_x_ overlayer and metallic Ru work synergistically to enhance the performance. Although CO bond cleavage on the Ru (001) surface presents a high barrier of 2.15 eV, the presence of a reduced TiO_3_ cluster on the Ru (001) surface (TiO_3_/Ru(001)) lowers this barrier to 1.62 eV. This illustrates how optimized SMSI encapsulation can enhance catalytic activity by reducing activation barriers and fine‐tuning metal‐support interactions.

SMSI plays a pivotal role in influencing the selectivity of catalytic reactions. The selectivity of CO_2_ hydrogenation on a Ru/TiO_2_‐A catalyst undergoes significant changes depending on the presence or absence of SMSI.^[^ ^93^ ^]^ When the catalyst was reduced at 150 °C, no SMSI was formed, and CO_2_ hydrogenation produced exclusively CH_4_ through methanation, with formate (HCOO*) as the reaction intermediate (Figure 9b). However, upon increasing the reduction temperature to 300 °C, SMSI was induced, causing a complete shift in selectivity toward CO production via the reverse water–gas shift (RWGS) reaction, with carboxylate (COOH*) as the intermediate species. Remarkably, when this SMSI state was reversed by O_2_ treatment, the selectivity shifted back to CH_4_, underscoring the classical nature of C‐SMSI. These results demonstrate how SMSI alters surface intermediates and ultimately determines the final reaction product.

Metal encapsulation induced by SMSI effectively prevents the sintering of metal nanoparticles, significantly improving catalyst stability. This SMSI‐induced stabilization is particularly crucial for metals with low Tamman temperatures, such as Au (395 °C), which are more susceptible to sintering during the reaction than metals holding higher Tamman temperature like Ru (1089 °C). The importance of SMSI for catalyst stability becomes even more pronounced in reactions where stability, rather than selectivity, is the primary concern, such as CO oxidation to CO_2_. A notable example is the stability enhancement observed in Au/TiO_2_ catalysts during CO oxidation.^[^ ^32^ ^]^ Upon inducing SMSI by reduction at 400 °C, the Au/TiO_2_ catalyst showed no signs of sintering, maintaining stable performance at 300 °C over a 100 h reaction period (Figure 9c). In contrast, a Au/TiO_2_ catalyst lacking SMSI exhibited significant sintering (irreversible deactivation), leading to a gradual decline in activity over time. Thus, SMSI plays a crucial role in ensuring the long‐term stability of catalysts, particularly for sintering‐prone metals, thereby sustaining catalytic performance in stability‐critical reactions. Table 1 summarizes selected reactions catalyzed by metal/TiO_2_ under SMSI conditions, along with the corresponding conditions that induce SMSI.

**Table 1:** Summary of metal/TiO_2_ catalysts having SMSI in various chemical reactions.

Catalyst	Reaction	SMSI condition	Ref:
Pt/TiO_2_‐A	Toluene oxidation	In situ@250°C	[94]
Pt/TiO_2_‐A	m‐Cresol hydrodeoxygenation	Ex situ@350°C	[76]
Pd/TiO_2_‐A	Alkadienes (C_10_–C_13_) hydrogenation	Ex situ@200°C	[68]
Rh/TiO_2_‐R	Propane steam reforming	Ex situ@400°C	[73]
Pt/TiO_2_‐P25	Methane combustion	Ex situ@500°C	[75]
Pt/TiO_2_‐A	Methanol steam reforming	Ex situ@450°C	[82]
Rh/TiO_2_‐P25	CO_2_ hydrogenaion	In situ@250°C	[22]
Pd/TiO_2_‐A	Phenylacetylene hydrogenation	Ex situ@500°C	[83]
Ni/TiO_2_‐A	Guaiacol hydrodeoxygenation	Ex situ@400°C	[86]
Pd/TiO_2_‐A	2‐Propanol dehydrogenation	In situ@191°C	[91]
Pt/TiO_2_‐(A + R)	Nitrobenzene hydrogenation	NaBH_4_ reduction	[21]
Ni/TiO_2_‐R	Ethanol steam reforming	Ex situ@500°C	[70]
Ru/TiO_2_‐P25	HMF hydrogenation	Ex situ@400°C	[95]
Ni/TiO_2_‐A	Glycerol steam reforming	Ex situ@500°C	[96]
Pt/TiO_2_‐A	Formaldehyde oxidation	Ex situ@400°C	[97]
Au/TiO_2_‐P25	3‐Nitrostyrene hydrogenation	Ex situ@500°C	[98]
Ru/TiO_2_‐R	Polyethylene hydrogenolysis	Ex situ@500°C	[99]
Rh/TiO_2_‐P25	Syngas to ethanol	Ex situ@300°C	[100]
Pt/TiO_2_‐A	Nitrobenzothiazole hydrogenation	Ex situ@200°C	[101]
Cu/TiO_2_‐A	CO_2_ to CH_3_OH	Ex situ@300°C	[102]

### 7.2 Unraveling SMSI Dynamics: Implications for Catalytic Activity and Metal Exposure

If SMSI is dynamic during catalysis, it prompts the question: is SMSI a prerequisite for catalysis rather than a direct participant? If so, SMSI may form a passive stabilization layer during catalyst preparation, beneficial for storage but removable to expose active metal atoms during catalysis. This dynamic nature is best explored under in situ/operando conditions using advanced microscopic and spectroscopic techniques focusing on the surface rather than the bulk. Selected studies will be discussed in this section to highlight the highly dynamic nature of SMSI.

**Figure 10:**
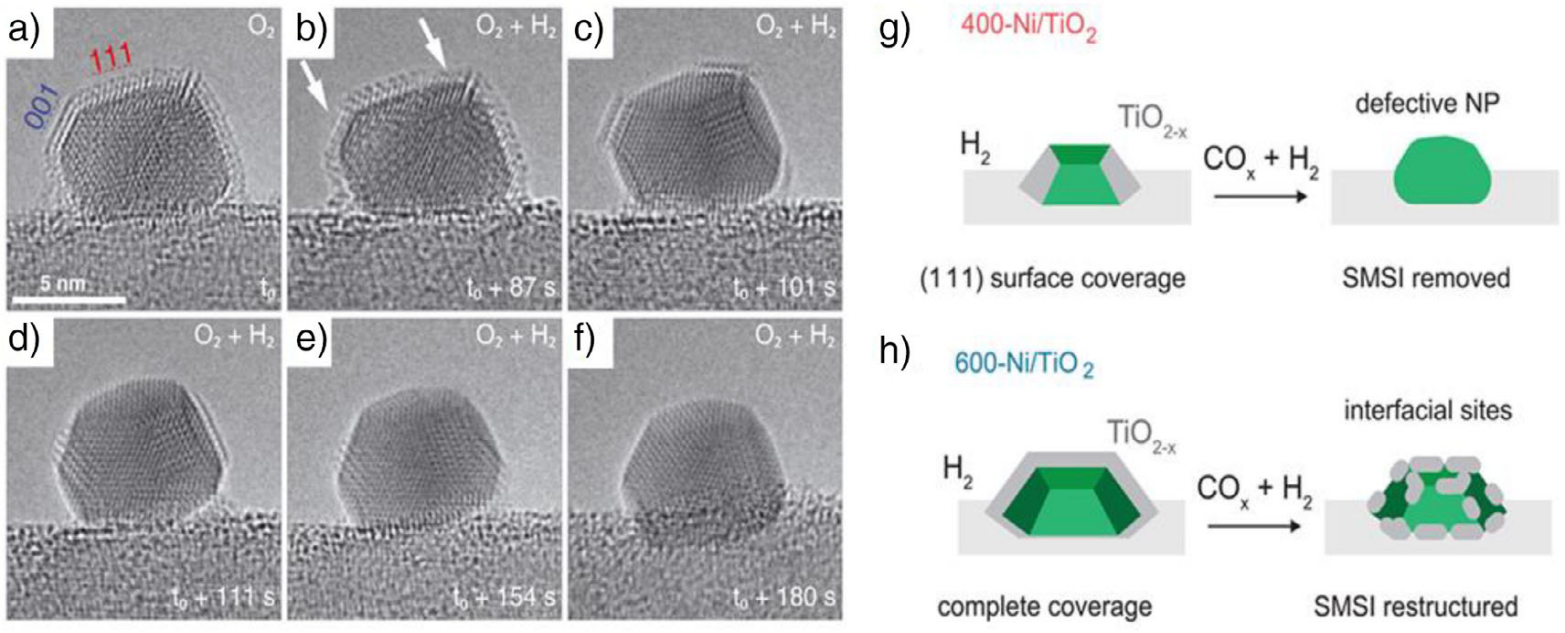
Dynamics of SMSI on TiO_2_ under reaction conditions. a–f) In situ TEM images of Pt/TiO_2_‐R catalyst under O_2_ + H_2_ atmosphere, showing time‐dependent retraction of TiO_x_, starting from the (111) plane followed by the (001) plane. Reprinted with permission from Ref. [103]. Copyright 2022, American Association for the Advancement of Science. g,h) Dependency of SMSI dynamics on the thickness of the encapsulation layer. g) Facet‐dependent encapsulation of Ni/TiO_2_‐P25 catalyst during 400 °C reduction, with subsequent de‐encapsulation under CO_2_ + H_2_ conditions. H) Partial retraction of SMSI formed on Pt/TiO_2_‐R catalyst at 600 °C reduction during CO_2_ hydrogenation conditions. Reprinted with permission from Ref. [105]. Copyright 2023, American Association for the Advancement of Science.

The dynamics of SMSI on a Pt/TiO_2_‐R catalyst using in situ TEM was investigated during the reaction of H_2_ + O_2_ to produce H_2_O, representing a combined oxidizing and reducing environment.^[^ ^103^ ^]^ An encapsulation layer was developed on Pt/TiO_2_‐R when heated at 600 °C in either H_2_ or O_2_ individually.^[^ ^104^ ^]^ However, when both gases were present simultaneously, the encapsulation layer disappeared. Specifically, when H_2_ was introduced to Pt/TiO_2_‐R where O_2_‐induced SMSI had already formed, H_2_ initiated the removal of oxygen from the TiO_x_ overlayer, destabilizing and eventually eliminating the SMSI layer. Mechanistically, changes in the overlayer began on the Pt (001) plane followed by retraction of the overlayer on the (111) plane (Figure 10a–f). Pt nanoparticles with their (111) planes perpendicular to the TiO_2_ (110) facet showed structural shifts, including twin plane formation and vertical motion. As the overlayer retracted from the (111) plane, slight rotations of the particle also occurred. These dynamics also impacted the TiO_2_ support, causing local structural collapse and rebuilding at the interface. Pt nanoparticles with their (111) planes parallel to the interface exhibited forward‐and‐backward motion, whereas the particles whose (001) plane parallel to the interface moved across the TiO_2_ surface. Upon returning the system to a pure O_2_ environment, the Pt nanoparticles changed their morphology from truncated cuboctahedra to spherical shapes, and the encapsulation layer reformed. Notably, the overlayer remained stable only in the presence of H_2_O, suggesting that it is the reactant gases (H_2_ and O_2_), rather than the product (H_2_O), that are responsible for the SMSI layer's removal. The findings suggest that while SMSI layers form under reductive or oxidative pretreatment, they retreat during catalysis, exposing active Pt sites and indicating the potential for SMSI removal in mixed reducing and oxidizing environments. This phenomenon may extend to other catalytic environments involving both reducing (H_2_ and for instance sacrificial reducing agents like isopropanol) and oxidizing (oxygen‐containing organic molecules), also reactions like partial oxidation of hydrocarbons, indicating that SMSI may be removed during catalysis.

The dynamics of SMSI under CO_2_ + H_2_ conditions under operando STEM was reported on a Ni/TiO_2_‐P25 catalyst.^[^ ^105^ ^]^ Upon reduction at 400 °C, Ni particles exhibited partial encapsulation, with the TiO_x_ layer showing facet‐dependent behavior – encapsulation was observed only on Ni(111). The interatomic Ti–Ti calculations indicated that the encapsulated layer was exclusively composed of a compressed rutile phase, despite the TiO_2_ (P25) containing both anatase and rutile phases. The introduction of CO_2_ along with H_2_ at 400 °C resulted in the complete removal of SMSI and further restructuring of the Ni particles (Figure 10g). This process was reversible in pure H_2_, which restored SMSI, underscoring the significant role of CO_2_ + H_2_ in SMSI dynamics. To explore the dynamics of a fully encapsulated Ni particle and a thicker TiO_x_ layer during CO_2_ + H_2_, the Ni/TiO_2_‐P25 was reduced at 600 °C. This higher temperature resulted in complete coverage of Ni particles by a TiO_x_ layer measuring 1–2 nm thick. When a CO_2_ + H_2_ mixture was introduced, the SMSI remained dynamic, however, resulting in only partial removal of the TiO_x_ layer, which left some Ni sections exposed (Figure 10h). This study also emphasizes that both the simultaneous presence of an oxidizing atmosphere (CO_2_) and a reducing atmosphere (H_2_), as well as the thickness of the TiO_x_ layer, play crucial roles in controlling SMSI dynamics during catalysis. Additionally, the thickness of the TiO_x_ layer can be manipulated through the reduction treatment or by varying the duration of that treatment.

Overall, these representatives in situ and operando studies under catalytic reaction conditions highlight the intricate relationships between metal‐support interactions through SMSI, reaction environments, and structural changes, offering a deeper understanding of how to optimize catalyst performance.

However, a comprehensive understanding of SMSI requires the use of advanced characterization techniques that provide insights into both the structural and electronic modifications of metal catalysts. These techniques enable precise identification of encapsulation layers, metal‐support interactions, and changes in oxidation states, helping to elucidate the underlying mechanisms governing SMSI formation and its impact on catalytic performance. Table 2 presents the key characterization techniques used for SMSI analysis, along with their descriptions and the specific SMSI insights they provide.

**Table 2:** Characterization techniques for SMSI: methods and key insights.

Technique	Description	SMSI insights
HRTEM	Provides high‐resolution imaging of individual metal nanoparticles.	Enables direct visualization of metal nanoparticle encapsulation layers due to SMSI.
HAADF‐STEM	Uses Z‐contrast imaging to differentiate between heavy metal particles and lighter encapsulating overlayers.	Allows for the identification of the encapsulation layer and helps in visualizing the interaction between the metal and support.
Electron energy loss spectroscopy (EELS)	Analyzes the energy lost by electrons interacting with a sample to examine the atomic structure and chemical composition.	Combined with HRTEM or HAADF‐STEM, EELS helps differentiate between the encapsulation layer and the metal nanoparticle by providing elemental composition details.
CO chemisorption	Measures the adsorption of CO molecules on metal surfaces, providing insights into metal dispersion.	A decrease in CO uptake indicates encapsulation. Calcination at higher temperature removes the SMSI layer and lower temperature reduction restores the CO chemisorption‐confirms C‐SMSI formation.
XPS	Measures binding energies of photoelectrons to analyze the surface composition and oxidation states.	A decrease in surface metal/support atomic ratios (e.g., metal/Ti ratio), indicates encapsulation. An increase in Ti^3^⁺/Ti⁴⁺ ratio provides evidence of partial reduction of the support during SMSI.
X‐ray absorption spectroscopy (XAS)	Includes XANES and EXAFS to study the absorption edges and fine structure of X‐ray absorption, providing information on oxidation states and coordination environments.	XANES identifies changes in the metal's valence state (absorption edge) due to SMSI. EXAFS reveals coordination number and bonding environment, helping to confirm structural changes at the metal‐support interface.
EPR	Detects unpaired electrons, particularly useful in identifying the presence of low‐valence states in reducible supports.	EPR signals at g = 1.90–1.99, corresponding to Ti^3+^, and at g ≈ 2.01, related to bulk oxygen vacancies in TiO_2_, serve as indicators of Ti reduction and oxygen vacancy formation during encapsulation. The intensity of these signals is directly related to the extent of SMSI, providing a means to monitor its progression.

## 8 Conclusion and Outlook

Titania‐supported metal catalysts are prevalent in heterogeneous catalysis. The SMSI in metal/TiO_2_ catalysts is known to have a substantial effect on their activity, selectivity, and overall stability. However, a comprehensive overview of how SMSI behaves on TiO_2_ with various metals and under different physical, chemical, and reactive conditions has yet to be consolidated. Understanding and modulating SMSI is crucial for designing efficient TiO_2_‐based catalysts for various applications. In this review, we provide a concise overview/guide of SMSI behaviors on TiO_2_ and modulation strategies to highlight their complexity. Analysis of SMSI across various metal/TiO_2_ catalysts has revealed a complex interplay of factors that influence these interactions. The in situ studies using TEM analysis have highlighted the evolution, progress, and reversibility of SMSI under reducing and oxidizing conditions. A key takeaway is the role of bulk oxygen vacancies and the surface free energy of the metals in driving SMSI processes. Furthermore, examining the electronic factors deepens the understanding of how charge transfer between the metal and TiO_2_ promotes metal encapsulation. Importantly, the correlation between alloy formation energy and SMSI strength/stability, and Ti affinity of metals enables a better prediction of SMSI strength and kinetics.

The crystalline phase and the morphology of TiO_2_ play a pivotal role in SMSI formation, with anatase and rutile phases exhibiting different behaviors. Different TiO_2_ morphologies expose various surface planes, resulting in varying degrees of SMSI on the distinct planes of anatase and rutile. Due to its lower stability, anatase TiO_2_ facilitates the migration of Ti^3+^ ions to metal nanoparticles, enhancing SMSI. In contrast, the greater thermodynamic stability of rutile TiO_2_ limits this interaction under similar conditions. Additional factors such as TiO_2_ particle size and surface modification with elements like barium or sodium further modulate SMSI, enabling additional tailoring for catalytic activity. Alternative strategies, such as ammonia exposure, wet‐chemical treatments, and low temperature reducing agents also offer new avenues to control SMSI while maintaining metal nanoparticle sizes. Notably, the in situ generation of SMSI during catalytic reactions highlights the importance of carbonaceous species from reactants in facilitating SMSI at much lower temperatures compared to C‐SMSI. Although these innovative nonclassical methods for modulating SMSI signify a noteworthy advancement in the field, C‐SMSI still stands out as a more straightforward and inherently less complex approach when it comes to practical applications.

**Figure 11:**
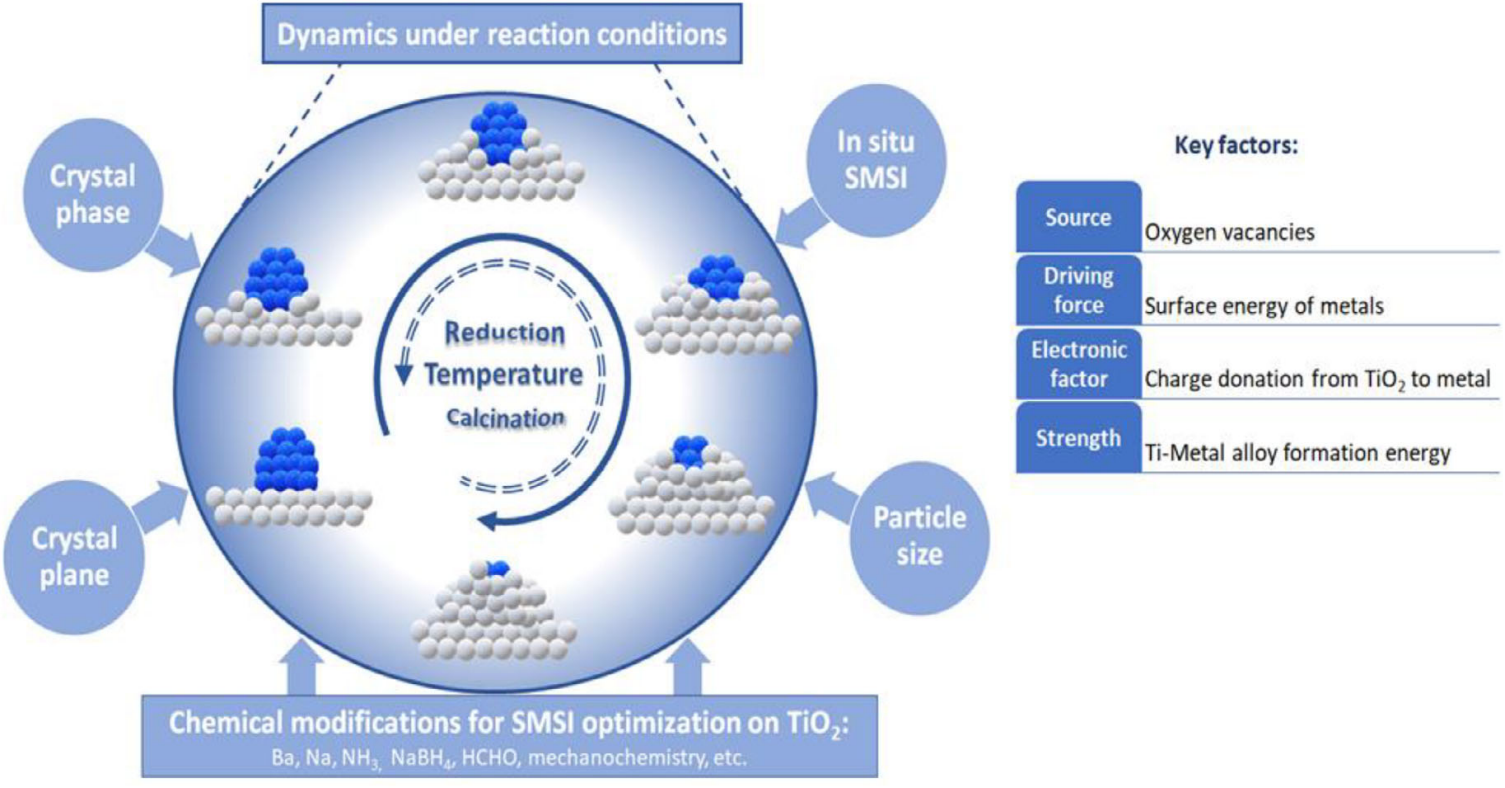
Summary of key findings on SMSI on TiO_2_.

The understanding of SMSI in metal/TiO_2_ systems reveals a critical gap regarding their dynamic behavior during catalytic reactions, despite the known pre‐SMSI benefits such as enhanced activity, selectivity, and stability. Studies have demonstrated that SMSI formation significantly optimizes metal‐support interactions. This optimization leads to improved catalytic activity, lower activation energy, and greater stability by preventing nanoparticle sintering. However, during catalysis, SMSI exhibits greater dynamics. For instance, whereas SMSI may act as a protective layer during catalyst preparation, its removal during catalysis can expose active metal sites. Different reaction environments, whether oxidizing or reducing, significantly affect the SMSI behavior. The reversibility of SMSI under certain conditions opens up possibilities for optimizing catalyst performance by carefully controlling the reaction environments. By offering a more detailed understanding of SMSI on TiO_2_ and its responsiveness to operational conditions, these insights pave the way for advancing catalytic technologies. Figure 11 provides a summary of key parameters and factors that induce and influence SMSI on TiO_2_.

Despite notable advancements in the understanding of SMSI in metal/TiO_2_ systems, the field is still in a state of active evolution. Recent studies have emphasized several key parameters influencing SMSI; however, it is expected that many additional factors will come to light, potentially altering our comprehension of SMSI's origin, strength, and dynamics. Furthermore, new methodologies for regulating SMSI are expected to be developed in the future. Continued research is crucial to gain deeper insights into SMSI dynamics in general under conditions that more closely mimic real‐world scenarios.

To guide SMSI research beyond the representative TiO_2_, in a more focused, standardized, and goal‐oriented direction, the following aspects are suggested for prioritization:

### 8.1 Standardizing SMSI Studies: A Framework for Consistent Evaluation

The complex nature of SMSI presents a challenge in reproducing this behavior consistently across different research groups. This hampers establishing a unified understanding of the SMSI mechanism and its effects on catalytic performance. To address this, a standardized methodology should be developed to evaluate SMSI effects on supported metal catalysts. This approach would include: 1) standardization of preparation protocols: Consistent methods for preparing catalysts, including metal loading, and the specific support phase and exposed planes used should be established. Detailed reporting of these procedures will enable other research groups to replicate experiments consistently and apply the catalysts for other relevant reactions. 2) Use of well‐defined characterization techniques: To better understand SMSI and compare results across studies, advanced characterization techniques such as in situ techniques (like XAS, in situ TEM, etc.) should be used. These techniques would allow researchers to observe changes in nanoparticle encapsulation, oxidation states, interface rearrangements, and morphology change directly. 3) Standard reaction conditions: Variability in reaction conditions can lead to differing outcomes. Researchers should agree on a set of standard conditions (e.g., reduction temperatures, reaction atmosphere, pressure, gas composition, etc.) for specific model reactions where SMSI is expected. This will help in comparing results across studies. 4) Collaborative database for SMSI studies: developing a shared, open‐access database where researchers can submit and compare their SMSI‐related findings would promote data accessibility. This database could include detailed information on experimental setups, material properties, and characterization results. The inclusion of negative results will also help in understanding the conditions under which SMSI is not reproducible, aiding in the identification of the critical parameters for successful observation. By implementing these strategies, it will be easier to develop a more consistent understanding of SMSI on supported metal catalysts.

### 8.2 Moving Beyond in Situ: The Need for Operando Studies to Accurately Assess SMSI in Catalytic Reactions

In situ studies provide valuable insights into SMSI dynamics under controlled conditions; however, they often fail to replicate the complex and variable environments encountered in actual catalytic reactions, such as fluctuating gas compositions, pressures, temperatures, and varying concentrations of reaction intermediates. These limitations can lead to misinterpretations of SMSI behavior and its impact on catalytic performance. For example, in situ studies typically utilize ultrahigh vacuum or controlled atmospheres, which do not accurately simulate practical conditions. This raises concerns about the relevance of their conclusions. To enhance the applicability of SMSI research, operando studies should be more widely adopted. Operando studies not only monitor the catalyst in real time under actual reaction conditions but also measure the catalytic activity simultaneously. This provides a more comprehensive and accurate understanding of the behavior of SMSI in practical scenarios. Operando techniques such as operando STEM, operando XAS, and operando infrared spectroscopy (IR) should be employed to simultaneously study the structural and chemical changes in SMSI under conditions closer to actual catalytic processes (e.g., higher temperatures, realistic pressures, and mixed gas atmospheres). These techniques allow researchers to track the evolution of SMSI during the catalytic cycle and correlate these observations directly with changes in catalytic activity. In operando studies, reaction parameters like temperature, pressure, and gas composition should also be dynamically varied to simulate industrial processes. This includes cycling between reducing and oxidizing environments, mimicking start‐up and shutdown conditions, and using complex reactant mixtures with intermediates or poisons. These conditions offer realistic insights into how SMSI behaves in fluctuating environments. A hybrid approach where in situ studies are complemented by operando measurements could bridge the gap between fundamental understanding and practical relevance. This approach could help refine the conclusions drawn from in situ experiments by contextualizing them within the broader, more complex operando data. Just as in situ studies benefit from standardized protocols, operando studies also require well‐defined guidelines. These should include standardized reaction conditions and common parameters for data collection. This would ensure that results from different research groups are comparable and can be reproduced. Moreover, advanced machine learning techniques could be applied to analyze the large datasets generated from operando studies, helping to identify subtle correlations between reaction conditions, SMSI evolution, and catalytic performance. By expanding the use of operando techniques, integrating them with in situ studies, and applying advanced data analysis, researchers can draw more relevant conclusions about SMSI and its impact on catalytic performance.

### 8.3 Temperature and Gas Composition: Key Players in the Stability of SMSI

Both reducing and oxidizing gases play crucial roles in catalytic processes. High concentrations of reducing gases, such as hydrogen (H_2_) or carbon monoxide (CO), can induce and strengthen SMSI. Conversely, oxidizing gases like oxygen (O_2_) or nitrogen oxides (NO_x_) can weaken or reverse the SMSI, which may lead to the redistribution of metal particles and alter their interaction with the support. These situations can result in rapid changes in SMSI behavior throughout catalytic cycles, making predictions and controls difficult. Additionally, exothermic or endothermic reactions can cause localized temperature variations on the catalyst surface. Higher temperatures can enhance the diffusion of support oxides over metal particles, leading to stronger encapsulation, whereas lower temperatures may inhibit this effect. Furthermore, rapid temperature changes can induce thermal stress on the catalyst, potentially resulting in particle sintering and structural rearrangements affecting SMSI. To mitigate the impacts of reactive gases and temperature fluctuations on SMSI, strategies must be developed to stabilize it, ensuring optimal catalyst performance under real‐world conditions. One effective approach to mitigate the impacts of reactive gases is to switch between reducing and oxidizing atmospheres, which can “reset” the SMSI state and help preserve catalyst activity. Controlled cycling of reaction conditions can be integrated into catalyst regeneration strategies. To prevent sintering and structural changes under elevated temperatures, modifying supports with high thermal stability, such as doped TiO_2_ or composite supports like TiO_2_‐SiO_2_, is one option. Additionally, introducing controlled defects into the support material can stabilize SMSI. For instance, defects on TiO_2_, can enhance metal‐support binding and reduce the likelihood of metal particle agglomeration and preserving SMSI at the individual particle level. Furthermore, integrating machine learning algorithms into reactor systems can predict when temperature spikes or gas composition changes are likely to occur and how they might affect SMSI. By analyzing historical reaction data, machine learning models can provide early warnings and suggest real time adjustments to maintain optimal SMSI and catalytic performance.

### 8.4 Stabilizing SMSI‐Addressing Impurity Challenges in Industrial Catalysis

In prolonged reactions, especially in continuous mode, the increasing concentration of impurities in the feed gas can influence SMSI strength. This effect is particularly pronounced in reactions like CO_2_ hydrogenation, a key process for sustainable fuel production, where industrial flue gases often contain impurities such as O_2_, SO_2_, H_2_S, NO_2_, and NH_3_. For instance, O_2_ and NO_2_ can oxidize both the metal (e.g., Pt or Pd) and the support, altering their oxidation states and potentially weakening or reversing SMSI. Sulfur‐containing impurities (SO_2_, H_2_S) can form stable sulfides on metal surfaces, affecting SMSI, whereas NH_3_ can produce surface nitrides impacting the SMSI. Despite their significance, the effects of these impurities on SMSI remain underexplored. Future research should prioritize operando studies to track SMSI evolution in the presence of such impurities under realistic reaction conditions. These investigations could yield valuable insights into the role of impurities in SMSI and catalytic performance, facilitating the development of more robust, efficient catalysts.

### 8.5 Exploring SMSI in Liquid‐Phase Catalysis

The limited application of SMSI in liquid‐phase reactions represents a significant gap in catalytic research. While SMSI has been extensively studied in gas‐phase systems, its behavior in liquid‐phase environments, particularly the influence of solvents and additives, remains poorly understood. Solvents with oxygen‐containing functional groups (e.g., water, alcohols, ethers, etc.) and inorganic additives like salts can substantially affect SMSI, either enhancing or hindering its effects. For example, water or alcohols may promote hydroxylation or hydration of the support, altering surface properties and potentially disrupting SMSI. In polar solvents, metal particles may agglomerate or detach from the support (leaching) due to solvation, resulting in a loss of SMSI. To close this research gap, systematic studies are needed to assess how different solvent classes (polar, nonpolar, protic, aprotic) influence SMSI. Moreover, investigating how common additives impact SMSI could enable the selection of components that either reinforce or disrupt SMSI, improving catalytic robustness. Ultimately, the underexplored behavior of SMSI in liquid‐phase reactions presents both a challenge in terms of in situ and operando characterization and a critical opportunity for future research. However, transferring the spent catalysts under inert conditions could be used as an initial strategy to study the SMSI effect in liquid‐phase catalytic reactions.

### 8.6 Bridging the Gap between Theory and Practice‐Enhancing SMSI Predictions for Real‐World Catalytic Conditions

Theoretical calculations, such as DFT, have been invaluable for understanding the mechanisms behind SMSI, offering detailed insights at the atomic and electronic levels. These computational tools, in general, shed light on how metals interact with supports, how surface modifications take place, and how these interactions influence catalytic behavior. However, much of the current theoretical work is based on idealized conditions, often overlooking the complexities of real‐world catalytic environments. As a result, these models may fail to fully capture the behavior of SMSI under practical conditions. Many theoretical studies simulate SMSI in vacuum or gas‐phase environments, neglecting the effects of solvents, additives, and temperature fluctuations typical of industrial processes. This disconnect can lead to discrepancies between theoretical predictions and experimental outcomes. For example, idealized models might miss the impact of solvents or contaminants on SMSI behavior, resulting in an incomplete understanding of how SMSI functions in practical applications. To improve the relevance and predictive power of theoretical models, key advancements are needed. By incorporating more realistic conditions‐such as temperature and pressure variations, as well as the presence of reactants, products, and impurities‐simulations can better align with experimental observations. Additionally, including the influence of common industrial contaminants like O_2_, SO_2_, H_2_S, NO_2_, and NH_3_ in simulations would further enhance the accuracy of theoretical predictions. By using DFT insights as a guide, researchers can design experiments that test SMSI behavior under specific conditions, such as different solvents or contaminants. Coupling these theoretical findings with experimental validation and machine learning‐driven screening could lead to the rational design of catalysts that exhibit effective SMSI in industrial environments.

As we continue to explore SMSI under different conditions, it becomes clear that a more meticulous approach is needed. Viewing SMSI as a dynamic interaction between the support, metal, and surrounding environment reveals new possibilities for optimizing catalytic reactions. It is anticipated that this review will inspire innovations in catalyst design, with precise control of SMSI being crucial for achieving targeted outcomes across various industrial processes.

## Conflict of Interests

The authors declare no conflict of interest.

## Data Availability

Data sharing is not applicable to this article as no new data were created or analyzed in this study.
